# Shotgun metagenomics and systemic targeted metabolomics highlight indole-3-propionic acid as a protective gut microbial metabolite against influenza infection

**DOI:** 10.1080/19490976.2024.2325067

**Published:** 2024-03-06

**Authors:** Séverine Heumel, Vinícius de Rezende Rodovalho, Charlotte Urien, Florian Specque, Patrícia Brito Rodrigues, Cyril Robil, Lou Delval, Valentin Sencio, Amandine Descat, Lucie Deruyter, Stéphanie Ferreira, Marina Gomes Machado, Adeline Barthelemy, Fabiola Silva Angulo, Joel. T Haas, Jean François Goosens, Isabelle Wolowczuk, Corinne Grangette, Yves Rouillé, Ghjuvan Grimaud, Marie Lenski, Benjamin Hennart, Marco Aurélio Ramirez Vinolo, François Trottein

**Affiliations:** a Univ. Lille, CNRS, INSERM, CHU Lille, Institut Pasteur de Lille, U1019 – UMR 9017 – CIIL – Center for Infection and Immunity of Lille, Lille, France; b Laboratory of Immunoinflammation, Institute of Biology, University of Campinas (UNICAMP), Campinas, Brazil; c Genoscreen, Lille, France; d Biomathematica, Rue des Aloes, Quartier Balestrino, Ajaccio, France; e Univ. Lille, CHU Lille, EA 7365 – GRITA – Groupe de Recherche sur les formes Injectables et les Technologies Associées, Lille, France; f Univ. Lille, INSERM, CHU Lille, Institut Pasteur de Lille, Lille, France; g Univ. Lrille, CHU Lille, Service de toxicologie et Génopathies, ULR 4483 – IMPECS – IMPact de l’Environnement Chimique sur la Santé humaine, Lille, France

**Keywords:** Influenza, gut microbiota, shotgun metagenomics, metabolomics, indole-3-propionic acid, disease severity

## Abstract

The gut-to-lung axis is critical during respiratory infections, including influenza A virus (IAV) infection. In the present study, we used high-resolution shotgun metagenomics and targeted metabolomic analysis to characterize influenza-associated changes in the composition and metabolism of the mouse gut microbiota. We observed several taxonomic-level changes on day (D)7 post-infection, including a marked reduction in the abundance of members of the *Lactobacillaceae* and *Bifidobacteriaceae* families, and an increase in the abundance of *Akkermansia muciniphila*. On D14, perturbation persisted in some species. Functional scale analysis of metagenomic data revealed transient changes in several metabolic pathways, particularly those leading to the production of short-chain fatty acids (SCFAs), polyamines, and tryptophan metabolites. Quantitative targeted metabolomics analysis of the serum revealed changes in specific classes of gut microbiota metabolites, including SCFAs, trimethylamine, polyamines, and indole-containing tryptophan metabolites. A marked decrease in indole-3-propionic acid (IPA) blood level was observed on D7. Changes in microbiota-associated metabolites correlated with changes in taxon abundance and disease marker levels. In particular, IPA was positively correlated with some *Lactobacillaceae* and *Bifidobacteriaceae* species (*Limosilactobacillus reuteri, Lactobacillus animalis*) and negatively correlated with *Bacteroidales* bacterium M7, viral load, and inflammation markers. IPA supplementation in diseased animals reduced viral load and lowered local (lung) and systemic inflammation. Treatment of mice with antibiotics targeting IPA-producing bacteria before infection enhanced viral load and lung inflammation, an effect inhibited by IPA supplementation. The results of this integrated metagenomic-metabolomic analysis highlighted IPA as an important contributor to influenza outcomes and a potential biomarker of disease severity.

## Introduction

A growing body of evidence suggests that the gut-to-lung axis plays a critical role in respiratory tract infections, including influenza A virus (IAV) infections (for reviews,^1–3^). Germ-free mice and mice chronically treated with broad-spectrum antibiotics are more susceptible to IAV infection and eventually develop severe and ultimately fatal disease.^4–8^ Mechanistically, various components of the gut microbiota (notably metabolites) diffuse into the bloodstream and remotely prime the lung to combat the virus by promoting interferon-dependent immunity (among other actions). Furthermore, IAV infection can alter the gut bacterial community, as demonstrated in animal models.^9–15^ These changes may have beneficial and/or detrimental consequences on the disease outcomes. It is possible that the blooming of some bacterial species (such as *Bifidobacterium animalis* and *Akkermansia muciniphila*) contributes to host resistance to IAV infection.^13,14^ We and others have shown that disruption of the gut microbiota in mice during IAV infection favors local and systemic secondary bacterial infections, both of which are major causes of death in humans.^10–12,16^ In humans, influenza also alters the composition of the gut microbiota, although the precise consequences on disease outcomes are yet to be defined.^17–20^ To date, changes in the gut microbiota during influenza have mostly been tracked using 16S rRNA gene sequencing. This approach has the limitation of low taxonomic resolution (i.e., up to the genus level), mainly due to a small amplicon size, and can be biased because of the hypervariable region targeted and the primer sequences used.^21–23^ In contrast, shotgun metagenomics allows for a more accurate estimation of changes in bacterial diversity and can identify bacterial species and sometimes even bacterial strains. In addition to taxonomic information, shotgun metagenomics provides important clues to the gene detection of gut microbiota, allowing the definition of the functional and metabolic potential of the microbiota community. To the best of our knowledge, the present study is the first to combine shotgun metagenomics, targeted systemic metabolomics, and measurements of influenza severity. We found several correlations among taxon abundance, gut microbial metabolites, and markers of disease severity. Notably, we identified the microbial tryptophan metabolite indole-3-propionic acid (IPA, the levels of which are markedly reduced during an IAV infection) as a potential biomarker of influenza resistance and as a target for microbiome-based therapeutic interventions.

## Results

### Influenza infection leads to a change in the microbiota’s composition, as assessed by shotgun metagenomic

Mice were infected with a sublethal dose of IAV, and body weight variations were measured during the course of infection (Supplementary Figure S1(a)). Body weight loss peaked at 17–18% of the initial body weight between days (D)8 and D10 post-infection (Supplementary Figure S1(b)). By D14, the mice regained some weight, but did not recover their starting weight (minus 3% of the initial weight). As expected, IAV infection was associated with a marked lung viral load and antiviral response at D7, while decreased expression of genes associated with epithelial integrity was observed (Supplementary Figure S1(c,d)). Infection was also associated with enhanced systemic concentrations of inflammatory cytokines and markers of an altered intestinal barrier, such as lipopolysaccharide-binding protein (LBP), at D7 (Supplementary Figure S1(e)).

**Figure 1.**
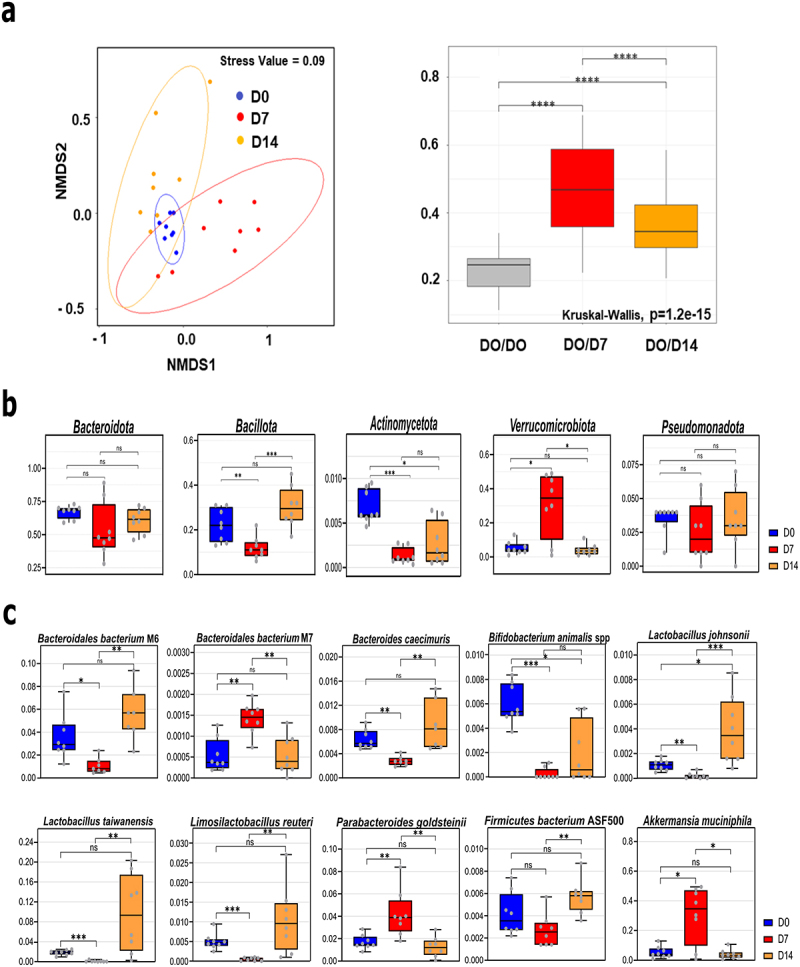
Altered composition of the gut microbiota during IAV infection. (a), *left panel*, Bray Curtis based nonmetric multidimensional scaling plot of all samples (stress value = 0.09). Each spot represents one sample and each group of mice is denoted by a different color (blue: non-infected/Day 0, red: D7, orange, D14). Distance between dots represents extent of compositional difference. *right panel*, Bray Curtis distance among samples at D0 (reference) and between each samples at D7 or D14 and D0. (b and c), relative abundance of taxa significantly different between D7 and D0 or D14 at the phylum (b), and species (c) level. Blue represents D0 samples; red represents D7; orange represents D14. Significant differences were determined using the Kruskal-Wallis test. Significant differences were determined using the Kruskal-Wallis test (* *p* < 0.05, ** *p* < .01, *** *p* < .001, **** *p* < .0001). NMDS, non-metric multidimensional scaling plot.

To examine changes over time in the gut microbiota during IAV infection, cecal contents from IAV-infected mice were collected at D7 and D14 post-infection, and extracted DNAs were subjected to shotgun sequencing. Non-infected mice sacrificed 7 days after PBS inoculation were used as controls (henceforth referred to as D0). First, we measured the α diversity index of each sample. A decrease in the number of species was observed on D7 and became significant on D14 (Wilcoxon, *p* < 0.05) (Supplementary Figure S2(a), *left* panel). Analysis of Shannon and Simpson indices indicated that α-diversity equitability was significantly and transiently diminished on D7 (Supplementary Figure S2(a), *middle* and *right* panels). The values on D14 were close to those observed on D0. This indicated that although the number of species was reduced on D14 (Supplementary Figure S2(a), *left* panel), the communities recovered their diversity evenness at this time point. Next, we determined the β-diversity by calculating the Bray-Curtis dissimilarity index between samples. The Bray-Curtis distances and non-metric multidimensional scaling of all samples revealed clustering at different time points, particularly on D0 (Figure 1a, *left* panel). An analysis of similarity and PERMANOVA showed that each group was significantly distinct from the others in terms of sample groupings and the corresponding R/R2 values (Table 1). Moreover, the D0 vs. D7 and D7 vs. D14 differences were greater than the D0 vs. D14 differences (*R* = 0.969 and 0.8295; R2 = 0.63357 and 0.47443 versus *R* = 0.2963 and R2 = 0.16761). The Bray-Curtis distances showed a drift in the cecal microbiota after infection, with a peak at D7 (Figure 1(a), *right* panel). The change in bacterial diversity on D14 was still statistically significant compared to that on D0. Taken together, these results show that IAV infection is associated with a slight (non-significant) and gradual decrease in bacterial richness and reduced community equitability (certainly bacterial dominance), which was most prominent on D7.

**Table 1.** Analysis of similarity (ANOSIM) and dissimilarity (PERMANOVA) with associated *p* values to test sample grouping (10,000 permutations).

	Statistical test	Statistic value	P value
3 groups	ANOSIM	*R* = 0.4567	0.0000999
	PERMANOVA	R^2^ = 0.50938	0.0000999
D0 vs D7	ANOSIM	*R* = 0.6211	0.00039996
	PERMANOVA	R^2^ = 0.38746	0.0013
D7 vs D14	ANOSIM	*R* = 0.5608	0.0014999
	PERMANOVA	R^2^ = 0.31484	0.0016
D0 vs D14	ANOSIM	*R* = 0.2963	0.0030997
	PERMANOVA	R^2^ = 0.16761	0.0042

Differential analyses from shotgun sequencing were performed for several taxonomic ranks, that is, from the phylum level to the species level. The relative abundances were used for each taxon at each rank. Wilcoxon’s test with Benjamini-Hochberg correction for parallel multiple testing showed significant changes in the relative abundance of bacterial phyla, families, and species during IAV infection. At the phylum level, the median relative abundance of Bacteroidota (previously known as Bacteroidetes, up to 70% at baseline) decreased transiently on D7, although the change was not statistically significant (Figure 1b). However, the relative abundance of Bacillota (previously known as Firmicutes) markedly changed over time. It decreased significantly from 22% of the identified bacterial taxa on D0 to 11% on D7. This decrease was transient because the relative abundance increased to 30% on D14. The reduced relative Bacillota frequency on D7 was mirrored by a marked but transient increase in the abundance of Verrucomicrobiota from 5% on D0 to 35% on D7. Notably, the relative abundance of Pseudomonadota (Proteobacteria) decreased transiently but not significantly on D7. Finally, the relative abundance of Actinomycetota (Actinobacteria) decreased significantly by D7 and remained low on D14. At a lower taxonomic level (family), the groups of animals differed with regard to *Muribaculaceae* (Bacteroidota), *Lachnospiraceae*, *Lactobacillaceae*, *Bifidobacteriaceae* (Bacillota) (drop at D7), and *Akkermansiaceae* (Verrucomicrobiota) (rise on D7) (Supplementary Figure S2(b)). At the species level, the relative abundances of four species of Bacteroidetes, four species of Bacillota, one species of Actinomycetota, and one species of Verrucomicrobiota significantly changed on D7 (Figure 1c). The relative proportions of the four Bacillota species (mostly *Lactobacillaceae* family), *Lactobacillus taiwanensis*, *Lactobacillus johnsonii*, *Limosilactobacillus reuteri*, and *Bacillota bacterium ASF500* decreased on D7 and rose on D14. The relative abundances of *Bacteroidales bacterium* M6 and *Bacteroides caecimuris* (*Muribaculaceae*) decreased, while those of *Parabacteroides goldsteinii* (*Tannerellaceae*, Bacteroidota) and *Bacteroidales bacterium* M7 (*Muribaculaceae*) increased on D7, and the four relative abundances appeared to have returned to baseline values by D14. The relative proportion of the Actinomycetota species *Bifidobacterium animalis* spp. strongly decreased during infection and did not return to its baseline value on D14. Finally, the relative abundance of *Akkermansia muciniphila* (a bacterium usually associated with good health, leanness, and fitness in humans^24^ increased markedly from 5% on D0 to 35% on D7 and returned to basal levels on D14. Linear discriminant analysis effect size (LEfSe) analyses indicated that *Akkermansia muciniphila*, *Parabacteroides goldsteinii*, and *Bacteroidales* bacterium M7 were among the species associated with changes at D7, whereas Lactobacillus species, *Bacillota bacterium ASF500* and *Bacteroidales bacterium* M1 and M6 were the most discriminant at D14 (Supplementary Figure S2(c)). Finally, the abundance of *Bifidobacterium animalis* spp. was a distinctive feature of the non-infected group.

### Influenza infection is associated with changes in the gut microbiota’s metabolic profile

**Figure 2.**
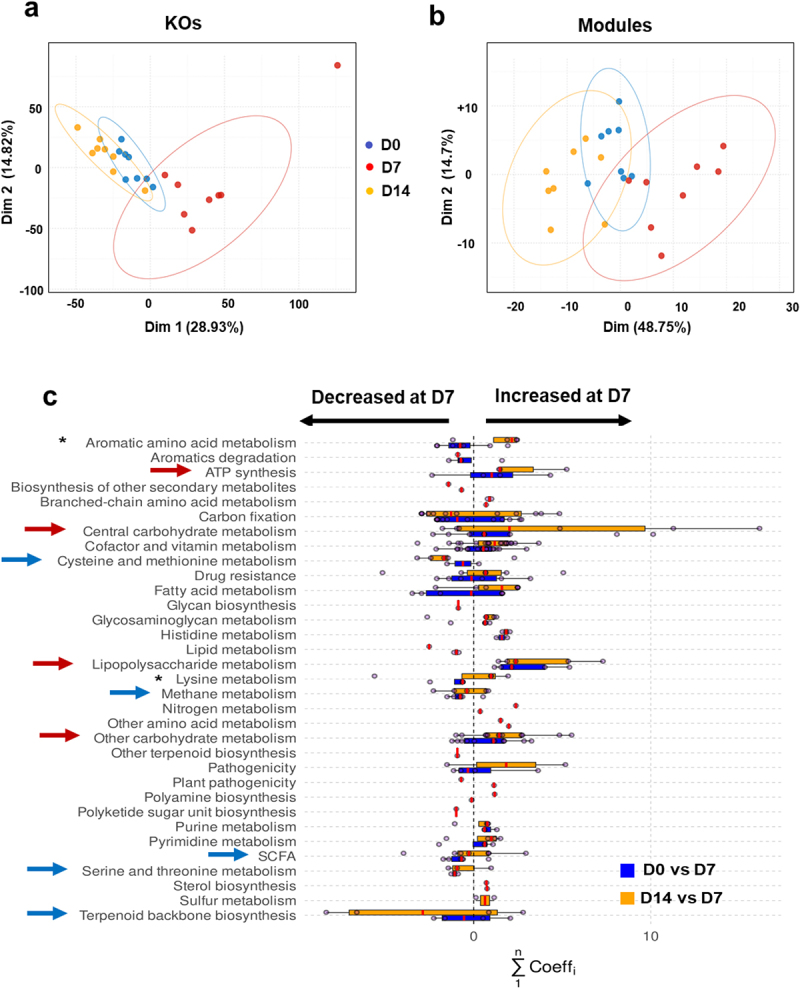
Functional profiles of the gut microbiota during IAV infection. (a and b), PCA of normalized relative abundances of KOs (a) and module scores (b). (c), representation at the category level of the KOs with the most varying songbird coefficients aggregated at the module level. For each module, the coefficients of the KOs taking part in it are summed up. Then, modules were grouped into categories according to larger biological functions. Each dot on the plot represents the sum of KO coefficients for a module. Red lines are medians and boxes represent Q1 and Q3. A positive coefficient means an increase of the module on D7, whereas a negative coefficient illustrates a decrease. The higher or lower the coefficient, the greater the change. Red arrows indicate increased categories and blue arrows indicate lowered categories. Asterisks denote categories decreased on D7 and increased on D14. The categories not shown have modules with KOs with null abundance (not detected) or not selected (not in the top 25% of the KOs with the most extreme coefficients).

We next analyzed functional markers in the gut microbiota of IAV-infected mice. To this end, we used KEGG annotations (generated by KEGG KofamScan^25^ in a gene-centric analysis of unassembled metagenomes. Principal component analysis (PCA) was performed on the normalized KEGG orthology (KO) relative abundance (Figure 2(a)). These projections tended to corroborate the conclusions of the taxonomic analyses (Figure 1(a)). The D7 group was well separated from the other two groups in the first dimension, which explained most of the variance (28.93%). The confidence ellipses around D0 and D14 overlapped substantially. This suggests that IAV infection induced a transient change in the functional profile on D7 although some differences persisted on D14. This is consistent with the community-based Bray-Curtis distances shown in Figure 1a. Next, we examined whether PCA of KEGG module scores would confirm the above observations. Modules regroup KOs into functionally meaningful biological reactions, providing more relevant insights into metabolic reaction potential. Differences between the functional profiles of IAV-infected and non-infected (D0) mice were still visible (Figure 2(b)). Overall, the confidence ellipses were wider (particularly for the D14 group), suggesting greater inter-individual variability. To assess the significance of the differences between experimental groups, we performed a PERMANOVA of the KO relative abundances and module score levels; both were significant and accounted for a high proportion of the variance (*p* < 0.001 for both; R^2^ = 0.49 and 0.61, respectively). Taken together, our results showed that IAV-infected mice (on D7) displayed an inherently different profile compared to non-infected mice (D0) and convalescent mice (D14) at both the KO and module levels.

We next searched for KOs with the greatest between-group variance in their relative abundances, which would explain the previously observed differences (Figure 2(c)). Three models (one for each pair of groups) were constructed using the songbird algorithm^89^. However, only two settings were scrutinized, namely D0 vs. D7 and D7 vs. D14, the D0 vs. D14 comparison being ineffective at capturing true differences between the groups. Qurro’s log ratios were computed for each model coefficient. In each case, the log ratio differences were smaller for D0 versus D14 and greater for D0 versus D7 and D7 versus D14. 25% of the highest coefficients and 25% of the lowest coefficients set by the songbird algorithm were then summed at the (metabolic) module level (as described in the Materials and Methods section). These accounted for 542 and 506 of the highest coefficients and 460 and 495 of the lowest coefficients for D0 versus D7 and D7 versus D14, respectively. Of the 159 most-varying modules, 108 were present in both D0 vs. D7 and D7 vs. D14 models, which also partly explains the log ratio differences in the models discussed above. Most of the modules [72 out of 137 (53%) for D0 vs. D7, and 85 out of 130 (65%) for D7 vs. D14] and their associated categories had a sum of coefficients above 0, which was greater in the D7 group. Figure 2(c) shows that the categories shifted in the same direction in both the models. This highlights the tendency of the functional profiles to return to a D0-like “baseline” state on D14. Interestingly, the categories that had increased the most on D7 were “ATP synthesis”, “carbohydrate metabolism”, and “lipopolysaccharide metabolism” (red arrows in Figure 2(c)). In contrast, the categories that had decreased the most were “cysteine and methionine metabolism”, “methane metabolism”, “short-chain fatty acid (SCFA) metabolism”, “serine and threonine metabolism”, and “terpenoid backbone biosynthesis” (blue arrows, Figure 2(c)). Differences between D0 and D7 and D7 vs. D14 were noted in the categories “aromatic amino acid metabolism” (*i.e*. tryptophan) and, to a lesser extent, “lysine metabolism” (lower on D7 and higher on D14) (asterisks, Figure 2(c)). In summary, shotgun sequencing results indicated that IAV infection modulated the functional activity of the gut microbiota.

### Influenza is associated with altered abundance of genes involved in metabolic pathways

**Figure 3.**
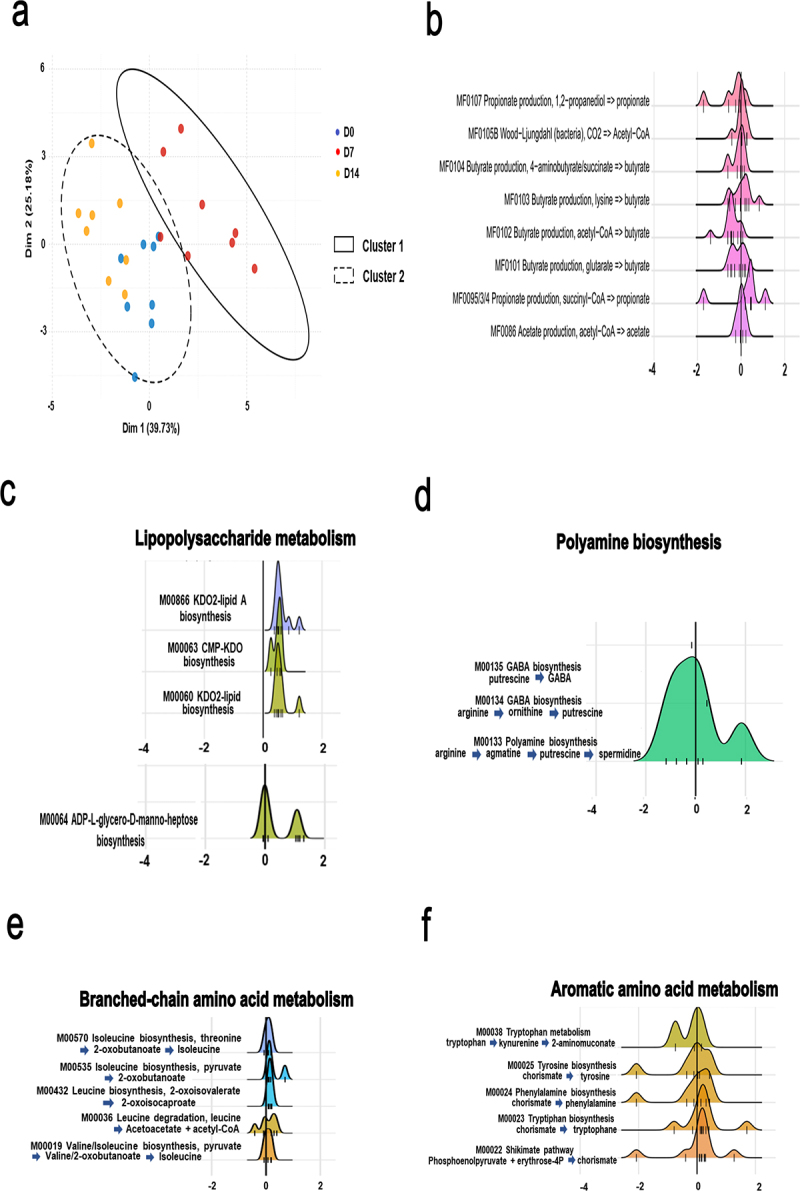
Alteration of specific metabolic compartments during influenza as assessed by shotgun analysis. (a and b), alteration of the abundance of genes involved in SCFA synthesis. (a), PCA of normalized relative abundances of modules covering SCFAs metabolism. (b), density ridgeline plots of the songbird coefficients of gomixer modules involved in SCFA synthesis and degradation. A positive coefficient is associated with an increase of the module’s KOs in D7, whereas a negative coefficient is associated with a decrease. The coefficients/differentials of the KOs computed by songbird are plotted on the x-axis (thin bars). The thick bar is the origin of the x-axis. (c–f), density ridgeline plots of the songbird coefficients of gomixer modules involved in LPS (c), fatty acid (b), polyamine (c), amino acid (arginine, proline, serine, threonine, cysteine and methionine) (d), branched- amino acid (d), branched chain amino acid (e), and aromatic amino acid (f) synthesis and degradation. A positive coefficient is associated with an increase of the module’s KOs in D7, whereas a negative coefficient is associated with a decrease. The coefficients/differentials of the KOs computed by songbird are plotted on the x-axis (thin bars). The thick bar is the origin of the x-axis.

Short-chain fatty acids (SCFAs) are derived from the fermentation of dietary fibers and carbohydrates by anaerobic bacteria. They play a key role in influenza infection.^12,16,26^ Several bacterial enzymes are critical for their production, notably acetate, propionate, and butyrate. We searched for genes encoding enzymes involved in SCFA biosynthesis and degradation, which led to the identification of 18 modules. As depicted in Figure 3(a), PCA analysis of these 18 modules clearly indicated a shift in the D7 group relative to the D0 and D14 groups (k-means clustering*, *p* < 0.01, PERMANOVA). Some modules relevant to SCFA production are shown in Figure 3(b). Four modules (MF0105A, MF0105B MF0086, and MF0075) leading to acetate production had at least one KO with a non-null coefficient (D0 vs. D7 and D14 vs. D7). These modules were not altered on D7 (Figure 3(b) and not shown). For propionate, four possible modules were scrutinized: production from succinate through propionyl-CoA and 2-methylmalonyl-CoA (MF0095/3/4), and via an alternative route (MF0084, which covers propionyl-CoA:succinyl-CoA transferase) from lactate (MF0106) and propanediol (MF0107). Only three were captured by the models, the module MF0084 had only null coefficients (Figure 3(b) and not shown). Both models reported a decrease in the abundance of the propanediol module (MF0107) on D7, but no clear behavior for the lactate (MF0106) module was observed. In contrast, succinyl-CoA (MF0095/3/4) module was augmented. All possible modules resulting in butyrate synthesis were detected by the models. We observed a clear decrease in the MF0102 (acetyl-CoA) and MF0101 (glutarate) modules at D7 in both models (Figure 3(b). For the 4-aminobutyrate/succinate (MF0104) and lysine (MF0103) modules, no clear behavior was observed (a tendency to decrease for MF0104). Collectively, the expression of bacterial enzymes involved in SCFA metabolism, more specifically those involved in butyrate production, was transiently altered during IAV infection.

Next, we focused on modules in the other altered functional categories (Figure 2(c)). The modules with the strongest differential (with most KOs moving away from zero) are shown in Figure 3(c–f) and Supplementary Figure S3. The most increased metabolic pathway in infected mice on D7 was “lipopolysaccharide metabolism” (Figure 3c). It is noteworthy that several modules involved in lipid metabolism (“fatty acid metabolism” and “lipid metabolism”) were either stronger (MF00873, M00874, M00083, M0082, M00093, M00089) or less strongly (M00087) abundant on D7 in both models (Supplementary Figure S3(a)). Bacterial carbohydrate metabolism provides energy and precursors for several biosynthetic pathways. Interestingly, modules involved in “carbohydrate metabolism” were also identified during infection (Supplementary Figure S3(b) and not shown). Modules involved in the generation of intermediates, such as oxaloacetate, oxaloglutarate, ribose- and ribulose-5 phosphate, and others, had coefficients centered around 0. Interestingly, the module involved in the biosynthesis of the polyamine spermidine (MF00133) was more abundant (Figure 3d), whereas the opposite effect was observed for modules involved in methanogenesis (Supplementary Figure S3(c) and not shown). With regard to amino acid metabolism, M00029, M0084, M00845 (covering the synthesis of arginine and the urea cycle more broadly), and M00879 (degradation of arginine to glutamate) were mostly upregulated in IAV-infected mice (Supplementary Figure S3(d), *left* panel). The abundance of modules involved in serine and threonine synthesis tended to be lower on D7, whereas the module involved in the degradation of serine to cysteine was more abundant (Supplementary Figure S3(d), *middle* and *right* panel). Isoleucine biosynthesis, leucine synthesis, and degradation modules were upregulated (“branched-chain amino acid metabolism”, Figure 3e). The valine metabolism was unaffected (data not shown). Lastly, most of the aromatic amino acid modules (including tryptophan synthesis and degradation, tyrosine synthesis, and phenylalanine synthesis) were affected on D7, as was the critical shikimate biosynthesis pathway (M00022, for the production of chorismate, a precursor of tryptophan, phenylalanine, and tyrosine) (Figure 3f). The modulation of M00038 (downregulated) and M00023 (mostly upregulated) suggests that tryptophan metabolism was altered during infection. Other pathways were affected by IAV infection, including those leading to the metabolism of cofactors and vitamins (data not shown). Overall, IAV infection was associated with many changes in the expression of microbial metabolism genes; these changes might influence the outcome of influenza.

### Influenza is associated with changes in the concentrations of microbial-associated metabolites

Next, we compared the concentrations of microbial-associated metabolites between the non-infected and IAV-infected mice. Given the key role of the gut-lung axis in influenza,^3^ we mostly focused on the serum metabolome because microbial metabolites must diffuse into the blood before they can affect lung functions. As the SCFA assay requires a large volume of serum, we measured SCFAs in the cecum. We analyzed the small-molecule metabolites produced by the gut microbiota, including SCFAs, tryptophan metabolites, polyamines, secondary biliary acids, and branched short-chain fatty acids. We also analyzed categories known to be modified by the host but from metabolites generated by the gut microbiota, such as short-chain acylcarnitine and trimethylamine N-oxide. Finally, we considered metabolites known to be used by the gut microbiota, such as dietary amino acids and branched-chain amino acids.

**Figure 4.**
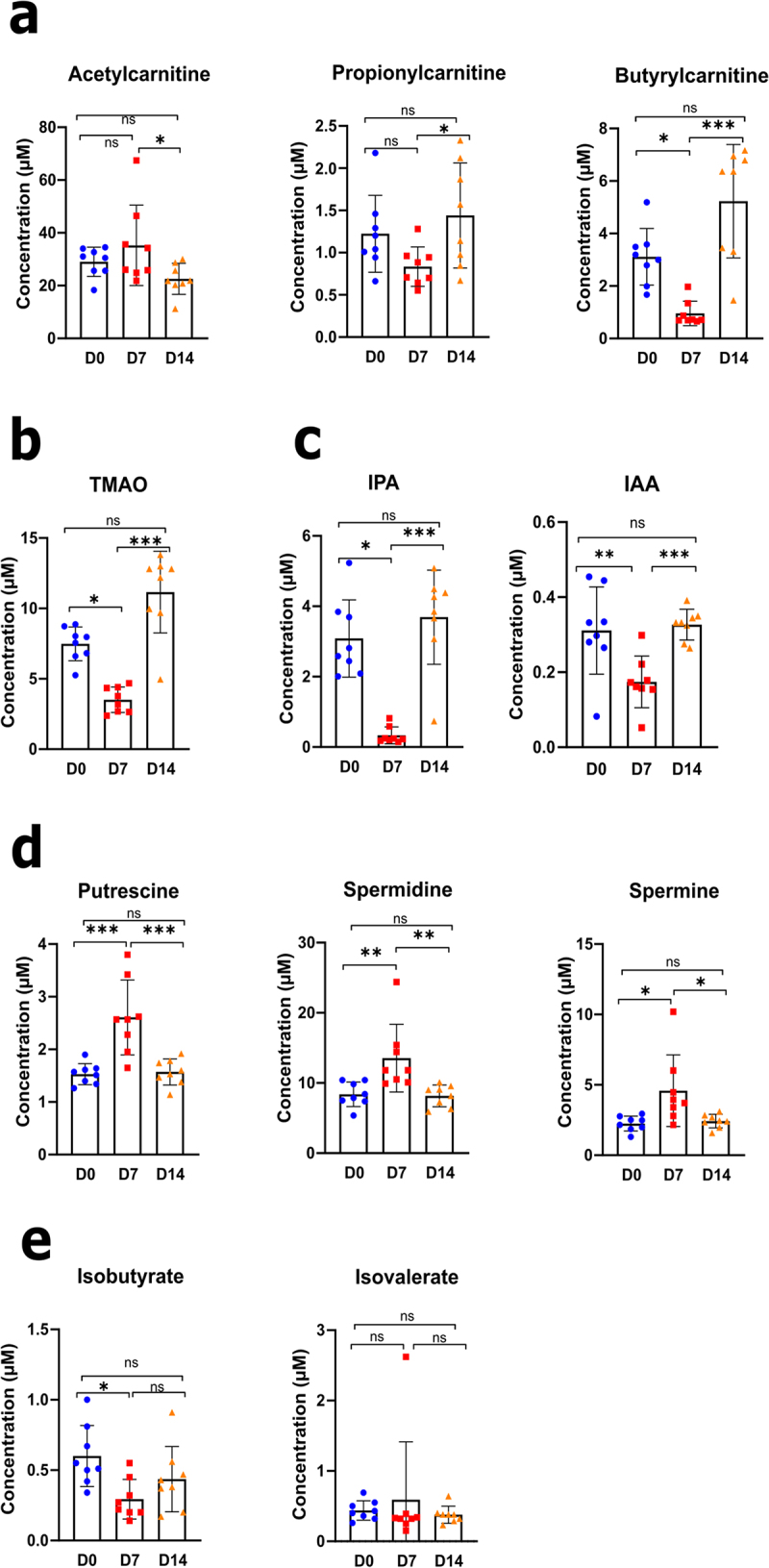
Alteration in blood metabolite production during an IAV infection. (a–e), L-carnitine and short-chain acetylcarnitines (a), TMAO (b), indole derivative (c), polyamine (d), and branched short-chain fatty acids (e) were measured in blood samples from each animal and at each time point, using targeted quantitative metabolomics (mean ± SD). Values for individual animal are presented (*n* = 7–8/time point) (µM). Significant differences were determined using one-way ANOVA test followed by Tukey’s multiple comparison test to parametric data. Nonparametric data was analyzed by Kruskal – Wallis ANOVA with Dunn’s posttest (*p < .05, **p < .01, ***p < .001). TMAO, trimethylamine N-oxide; IPA, indole-3-propionic acid; IAA, indole-3-acetic acid.

As shown in Supplementary Figure S4 and in line with our previous results and those from others,^12,27^ influenza was associated with a significant decrease in the cecal concentrations of SCFAs, including acetate, propionate, and butyrate. The short-chain acylcarnitines acetylcarnitine (C2), propionylcarnitine (C3), and butyrylcarnitine (C4) are derivatives of acetate, propionate, and butyrate, respectively. Relative to D0, the blood concentrations of propionylcarnitine and butyrylcarnitine were lower on D7 (although not significantly for propionylcarnitine), and that of acetylcarnitine was lower on D14 (Figure 4(a)). Notably, the blood concentration of acylcarnitine was stable on D7 and fell on D14. The gut microbiota can transform various dietary nutrients (e.g., choline and l-carnitine) into trimethylamine, which is subsequently oxidized to trimethylamine N-oxide (TMAO) by liver enzymes.^28^ Relative to D0, the concentration of TMAO decreased significantly on D7 but recovered on D14 (Figure 4(b)). These data suggest that IAV infection influences the trimethylamine-forming gut bacteria. The dietary amino acid tryptophan can be metabolized into various indole-containing metabolites by gut bacteria via tryptophanase. Interestingly, the blood concentration of indole-3-propionic acid (IPA), a major indole-containing tryptophan metabolite produced by the commensal microbiota,^29^ decreased dramatically by D7 and regained steady-state levels by D14 (Figure 4(c)). To a lesser extent, the concentration of indole-3-acetic acid (IAA), another microbiota-derived tryptophan metabolite, reduced on D7. Given that circulating tryptophan levels were not strongly affected during infection (Supplementary Figure S5(a)), the changes in IPA indicated that the microbial tryptophan-indole pathway was altered during IAV infection (Figure 3f). Polyamines (including putrescine, spermidine, and spermine) are produced by the gut microbiota from arginine as part of the urea cycle. As shown in Figure 4d and in line with Figure 3d, the concentrations of putrescine, spermidine, and spermine were transiently elevated on D7. Primary bile acids (produced by the liver as conjugates) are transformed into secondary bile acids by gut microbiota.^30^ The levels of secondary and primary bile acids did not change during IAV infection, suggesting that this pathway was unaffected (Supplementary Figure S5(b)). The gut microbiota modulates branched-chain amino acid levels in the blood because it can both produce and consume them. Blood concentrations of the branched-chain amino acids valine, leucine, and isoleucine did not vary significantly during IAV infection (Supplementary Figure S5(c)). Branched amino acids are fermented by the gut microbiota into isobutyrate and isovalerate, i.e. branched SCFAs.^31^ As shown in Figure 4e, the cecal concentration of isobutyrate significantly decreased on D7, whereas that of isovalerate remained constant. Similarly, gut microbiota can decrease the availability and metabolism of some dietary amino acids (such as tryptophan and arginine) by increasing the production of microbial proteins and various metabolites. The level of arginine (but not tryptophan) tended to decrease transiently during infection (Supplementary Figure S5(a)). Taken together, these data fit with the apparent functional (metabolic) alterations in the gut microbiota revealed by the shotgun sequencing analysis. Rearrangements in these microbial metabolic pathways may influence influenza outcomes.

### Alterations in taxon abundance and microbiota-associated metabolites are correlated with markers of influenza severity

**Figure 5.**
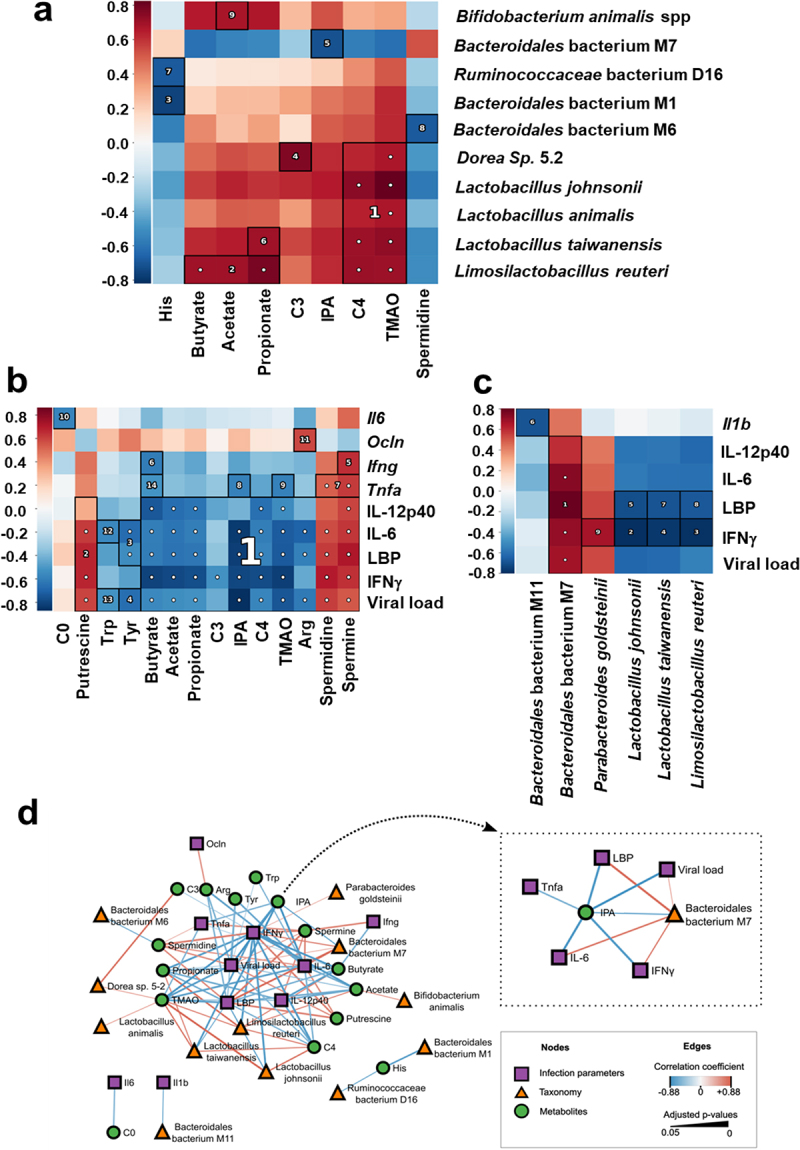
Associations between taxonomic and metabolomic features from non-infected and IAV-infected animals. (a), Hallagram representation for metabolites associations with microbial taxa. Metabolites shown in figure 6 were analyzed. Spearman correlation was used as similarity metric, with negative values represented in blue and positive values represented in red. Significant blocks were numbered in descending order of significance. (b and c), correlations between bacterial metabolites (b) and taxa (c) and infection-related variables. a–c, only taxa differentially represented between mock-infected and IAV-infected mice were taken into account. The false discovery rate (FDR) was controlled using the Benjamini-Hochberg method with alpha 0.05 and the expected false negative rate (FNR) for block associations was the default 0.2. (d), network of correlations between metabolites, taxa, and infection parameters. The shape and color of nodes indicate the group of data (metabolites, taxa, or infection parameters). Edges colors indicate the correlation coefficient (blue for negative and red for positive), whereas edge thickness is proportional to significance (thicker lines indicate lower adjusted p-values). Only associations with adjusted p-value ≤0.05 and correlation coefficient ρ ≥ |0.15| were included. *Inset*, Subnetwork of direct associations with IPA. Arg, arginine; his, histidine; Tyr, tyrosine; Trp, tryptophan; C0, carnitine; C3, propionylcarnitine; IPA, indole-3-propionic acid; C4, butyrylcarnitine; TMAO, trimethylamine N-oxide.

We next investigated the significance of the differences in taxa abundance and bacterial metabolite levels between non-infected and infected (D7 and D14) animals. Putative associations between taxonomic and metabolomic features were evaluated using pairwise Spearman correlation tests, hierarchical clustering, and the identification of densely associated blocks on a heatmap (Figure 5(a)). The statistically significant blocks included SCFAs (butyrate, acetate, and/or propionate), which were positively correlated with *Limosilactobacillus reuterii* (block 2), *Lactobacillus taiwanensis* (block 6, propionate only), and *Bifidobacterium animalis* spp. (block 9, acetate only) (Figure 5(a) and Table 2). *Lactobacilli* species were also positively correlated with butyrylcarnitine (C4) and TMAO levels (block 1). Changes in the abundance of *Dorea Sp. 5–2* were positively correlated with the levels of propionylcarnitine (C3, block 4), butyrylcarnitine (C4), and TMAO (cluster 1). In contrast, histidine was negatively correlated with *Bacteroidales* bacterium M1 (block 3) and *Ruminococcaceae* bacterium D16 (block 7). Moreover, *Bacteroidales* bacterium M7 was negatively correlated with IPA (block 5) and *Bacteroidales* bacterium M6 was negatively correlated with spermidine (block 8). Finally, the *Lactobacillaceae* and *Bifidobacteriaceae* species *Limosilactobacillus reuteri, Lactobacillus taiwanensis*, *Lactobacillus animalis, Lactobacillus johnsonii* positively correlated with IPA.

**Table 2.** Significative clusters identified with HAllA for taxonomy and metabolomics data. Shifts in species abundances are indicated inside parenthesis.

Cluster rank	Cluster X	Cluster Y	Best adjusted p-value
1	*Dorea sp. 5–2* (down at D7) *Lactobacillus johnsonii* (down at D7) *Lactobacillus animalis* (down at D7) *Lactobacillus taiwanensis* (down at D7) *Lactobacillus reuteri reuteri* (down at D7)	C4; TMAO	0.001918
2	*Lactobacillus reuteri reuteri* (down at D7)	Butyrate (cecum); Acetate (cecum); Propionate(cecum)	0.008292
3	*Bacteroidales bacterium M1* (down at D7)	His	0.009811
4	*Dorea sp. 5–2* (down at D7)	C3	0.009811
5	*Bacteroidales bacterium M7* (up at D7)	3-IPA	0.026983
6	*Lactobacillus taiwanensis* (down at D7)	Propionate(cecum)	0.028723
7	*Ruminococcaceae bacterium D16* (down at D7)	His	0.03179
8	*Bacteroidales bacterium M6* (down at D7)	Spermidine	0.03179
9	*Bifidobacterium animalis* (down at D7)	Acetate (cecum)	0.037395

Next, we attempted to measure correlations between the gut microbiota composition, metabolite levels, and indices of influenza severity (body weight loss, viral load, and systemic and pulmonary markers of inflammation; Supplementary Figure S1). We found that several bacterial metabolites and (to a lesser extent) several gut microbiota taxa were strongly correlated with indices of influenza severity (Figure 5(b,c)). For instance, SCFAs, propionylcarnitine (C3), butyrylcarnitine (C4), IPA, TMAO, and some amino acids were negatively correlated with pulmonary viral load, while spermine, spermidine, and putrescine (all polyamines) were positively correlated with these markers (blocks 1, 2, 4, and 13) (Figure 5(b) and Table 3).

**Table 3.** Significative clusters identified with HAllA for taxonomy and infectious diseases parameters data. Shifts in species abundances are indicated inside parenthesis.

Cluster rank	Cluster X	Cluster Y	Best adjusted p-value
1	IL-12p40 IL-6 LBP IFNγ Viral load	*Bacteroidales bacterium M7* (up at D7)	0.0021
2	IFNγ	*Lactobacillus johnsonii* (down at D7)	0.0021
3	IFNγ	*Lactobacillus reuteri* (down at D7)	0.0021
4	IFNγ	*Lactobacillus taiwanensis* (down at D7)	0.004
5	LBP	*Lactobacillus johnsonii* (down at D7)	0.024
6	*Il1b*	*Bacteroidales bacterium M11* (down at D7)	0.033
7	LBP	*Lactobacillus taiwanensis* (down at D7)	0.033
8	LBP	*Lactobacillus reuteri* (down at D7)	0.043
9	IFNγ	*Parabacteroides goldsteinii* (up at D7)	0.048

The above-mentioned metabolites showed a similar trend toward a correlation with local inflammatory markers (transcripts) and systemic inflammatory markers (proteins). At the genus level, the lower relative abundance of *Lactobacilli* species *Limosilactobacillus reuteri*, *Lactobacillus taiwanensis* and *Lactobacillus johnsonii* was negatively correlated with lung viral load, systemic inflammation, and leaky gut markers (Figure 5(c) and Table 4). Similar (but weaker) correlations were observed for *Bacteroidales bacterium* M11 (*Il1b*). In contrast, *Bacteroidales* bacterium M7 and *Parabacteroides goldsteinii* were positively associated with viral load and systemic inflammation.

**Table 4.** Significative clusters identified with HAllA for infection parameters and metabolomics data.

Cluster rank	Cluster X	Cluster Y	Best adjusted p-value
1	IL-12p40 IL-6 LBP IFNγ Viral load	Butyrate Acetate Propionate C3 3-IPA C4 TMAO Arg Spermidine Spermine	0.005
2	IL-12p40 IL-6 LBP IFNγ Viral load	Putrescine	0.005
3	IL-6 LBP	Tyr	0.005
4	Viral load	Tyr	0.005
5	*Ifng*	Spermine	0.008
6	*Ifng*	Butyrate	0.013
7	*Tnfa*	Spermidine Spermine	0.013
8	*Tnfa*	3-IPA	0.016
9	*Tnfa*	TMAO	0.017
10	*Il6*	C0	0.017
11	*Ocln*	Arg	0.017
12	IL-6	Trp	0.020
13	Viral load	Trp	0.045
14	*Tnfa*	Butyrate	0.045

We integrated various correlations into the network. The viral load and inflammatory markers occupied central positions, indicating their links with many metabolites (including SCFAs, short-chain acylcarnitines, polyamines, some amino acids, IPA, and TMAO) (Figure 5(d)). In peripheral positions, some of the taxa (such as *Lactobacilli* and *Bacteroidales* species) were linked primarily to metabolites but also to markers of disease severity. The network contained some relatively disconnected components, such as the negative correlation between histidine on one hand and *Bacteroidales bacterium* M1 and *Ruminococcaceae bacterium* D16. Among the centrally positioned metabolites, IPA was negatively correlated with the viral load, inflammatory markers, and *Bacteroidales bacterium* M7 (Figure 5(d), *inset*). Overall, the changes in some gut microbiota taxa and metabolites correlated with the indices of influenza severity.

### Supplementation of IPA reduces the severity of influenza

**Figure 6.**
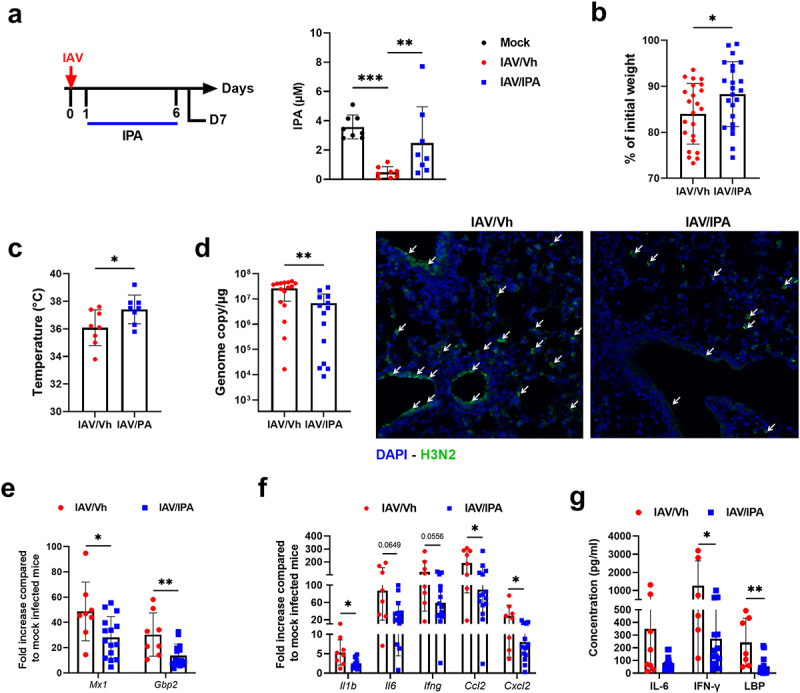
Effect of IPA treatment on viral load and inflammation during influenza infection. (a), *left* panel, schematic procedure. Mice were daily treated by oral gavage with vehicle (0.5% methyl cellulose in sterile water) or IPA (40 mg/kg/day) on D1 until D6. Mice were sacrificed on D7. *right* panel, systemic concentration of IPA in vehicle-treated and IPA-treated IAV-infected mice. Non-infected (mock) mice were used as controls. Errors indicate mean ± SD (*n* = 8). (b), percentage loss relative to initial body weight. Errors indicate mean ± SD (*n* = 22–24, pool of three independent experiments). (c), body temperature (*n* = 8). (d), *left* panel, quantification of viral load in the whole lung using specific TaqMan RT-qPCR. Data are expressed as genome copy number (M1 protein)/μg RNA. *right* panel, viral protein labelling (immunofluorescence) was performed on lung sections collected at 7D. Bars: 25 μm. (e and f), mRNA copy numbers were quantified by RT-qPCR. The data are expressed as the mean of change relative to average gene expression in non-infected animals. (g), serum proinflammatory cytokines and markers of altered intestinal barrier were quantified by ELISA. (d-g). Errors indicate mean ± SD (*n* = 7–16, two pooled experiments). Significant differences were determined using using the Kruskal-Wallis test (a) or the Mann Whitney *U* test (b-g) (*p < 0.05; ** *p* < .01, *** *p* < .001).

In view of the massive drop in IPA level on D7 (Figure 4(c)), its correlation with most of the infection parameters (Figure 5(d)), and the reported beneficial effects of this compound in many disease settings (ranging from inflammatory to metabolic disorders^32,33^), we hypothesized that IPA could be important during influenza. To do so, animals were supplemented with IPA (oral gavage, 40 mg/kg/day) or vehicle, as a control. Treatment started one day after IAV infection and then daily during infection (Figure 6(a), *left* panel). Animals were sacrificed at the peak of the acute inflammatory phase (day D7). Targeted liquid chromatography coupled with tandem mass spectrometry (LC-MS/MS) revealed that IPA treatment partially restored the systemic concentration of IPA in IAV-infected mice (Figure 6(a), *right* panel). IPA supplementation did not significantly alter gut microbiota’s composition during infection, as revealed by Bray Curtis-based PCA (Supplementary Figure S6a). LEfSe analyses revealed that IPA supplementation altered the relative abundance of few bacterial species (Supplementary Figure S6b). In term of clinical index, IPA supplementation significantly attenuated body weight loss due to infection (Figure 6(b)). This effect associated with a reduced decline in body temperature (Figure 6(c)), suggestive of the clinical benefit afforded by treatment. Interestingly, relative to vehicle-treated controls, mice treated with IPA showed a more rapid recovery, evident in both body weight and temperature regain (Supplementary Figure S6(c,d)). The potential effect of IPA supplementation on viral load was then assessed. Interestingly, IPA administration was associated with a significantly lower viral load in the lungs, as assessed by quantification of M1 protein transcripts in RT-qPCR assays (Figure 6(d), *left* panel). Immunofluorescence staining of lung sections confirmed a lower viral load in the IPA-treated animals (Figure 6(d), *right* panel). The mRNA expression of interferon-stimulated genes, such as *Mx1* and *Gbp2* (which is proportional to the viral load at D7), was lower in IPA-treated animals than in non-supplemented animals (Figure 6(e)). We then investigated the effects of IPA supplementation on lung inflammation. The levels of inflammatory gene transcripts (such as cytokines and chemokines: *Il1b*, *Il6*, *Ifng*, *Ccl2* and *Cxcl2*) were significantly lower in IPA-treated animals than in controls (Figure 6(f)). Treatment with IPA was also associated with lower serum levels of IL-6 and IFN-γ, recognized markers of systemic inflammation, as well as LBP, an indicator of altered intestinal barrier integrity (Figure 6(g)). Overall, IPA supplementation during the course of influenza partially reversed the detrimental disease outcomes (body weight loss, viral load and inflammation), which confirmed the results of the correlation analysis shown in Figure 5d.

### IPA-producing bacteria are important during influenza

**Figure 7.**
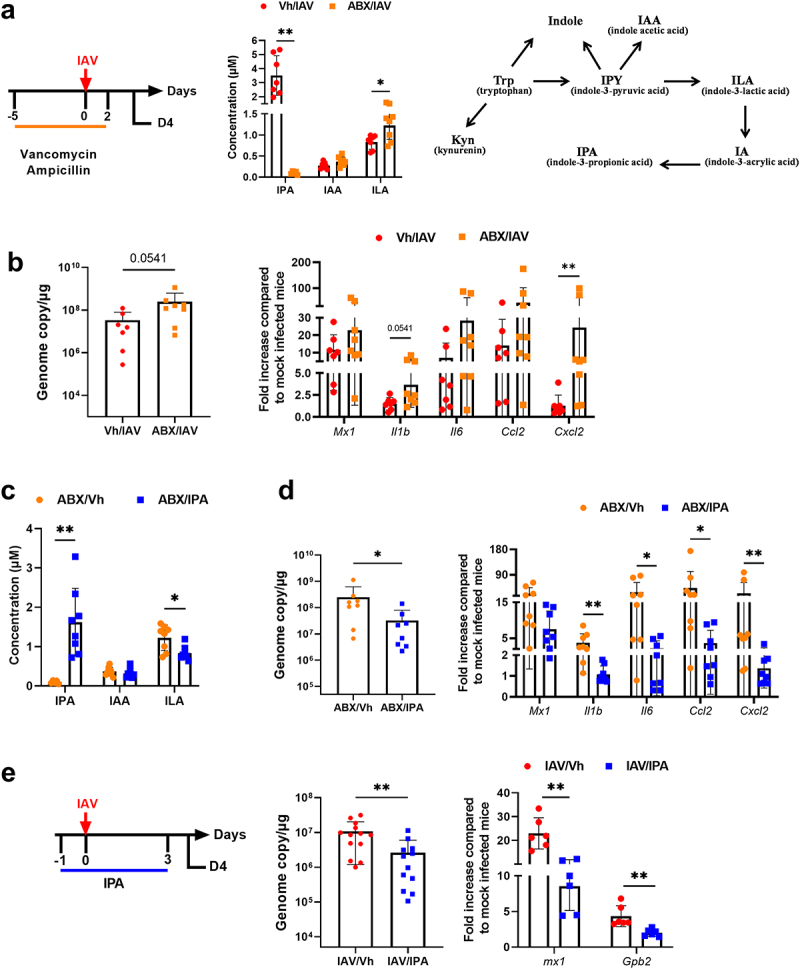
Role of IPA-producing bacteria in the control of viral load and inflammation. (a), *left* panel, schematic procedure. Mice were treated with vancomycin and ampicillin 5 days prior to the onset of infection until D2. Mice were sacrificed on D4. *middle* panel, systemic concentration of IPA, ILA and IAA in antibiotic-treated mice. *right* panel, schematic showing the conversion pathway of tryptophan into different bioactive indole metabolites including those that require the gut microbiota for conversion. (b), quantification of viral load in the whole lung using specific TaqMan RT-qPCR. Data are expressed as genome copy number (M1 protein)/μg RNA. (c and d), the same parameters (a and b) were measured by this time after supplementation, or not, with IPA (oral gavage, 40 mg/kg/day, from one day before infection to D3). (e), *left* panel, experimental procedure. Mice were daily treated or IPA one day before infection until D3. Mice were sacrificed on D4. *middle* and *right* panels, viral load and gene expression were quantified by RT-PCR. For all graphs, errors indicate mean ± SD. a-d and e, *right* panel, one experiment out of two is depicted (*n* = 6–8). e, *left* panel, a pool of two experiments is depicted (*n* = 13–14). Significant differences were determined using the Mann Whitney *U* test (*p < .05; ** *p* < .01).

IPA is produced exclusively by the microbiota.^29^ To confirm that the natural production of IPA by the gut microbiota is important in the control of influenza, mice were exposed to vancomycin and ampicillin 5 days prior to the onset of infection until D2. Mice were sacrificed on D4 (Figure 7(a), *left* panel). These antibiotics have been shown to target IPA-producing bacteria.^34,35^ As expected, antibiotic treatment profoundly altered the gut microbiota’s composition as revealed by LEfSe analysis (Supplementary Figure S7). In line with the above findings,^34,35^ targeted LC-MS/MS revealed that the systemic concentration of IPA was dramatically reduced upon antibiotic treatment (Figure 7(a), *middle* panel). In contrast, there was no change in IAA levels (Figure 7(a), *right* panel for the metabolic pathway). Furthermore, the circulating concentration of indole-3-lactic acid, which gives rise to the IPA precursor indole-2-acrylic acid, was unaffected by antibiotic treatment. We then assessed the effects of antibiotic treatment on viral load and lung inflammation. Compared with control mice, antibiotic-treated mice had a higher viral load and expressed enhanced levels of inflammatory cytokines in the lungs (Figure 7(b)). IPA supplementation in antibiotic-treated mice restored the systemic level of IPA (Figure 7(b)). Interestingly, this was associated with reduced viral load and inflammatory transcript levels in the lungs (Figure 7(d)). These data suggest that gut microbiota’s production of IPA reduces viral loads and mitigates inflammation during influenza infection. We then investigated whether IPA could function as a prophylactic factor against influenza under normal antibiotic-free conditions. To this end, mice were gavaged with IPA one day before infection and then daily during infection. As depicted in Figure 7(e), IPA treatment resulted in a lower virus load. Consistent with this, the expression of pulmonary ISGs was reduced. Collectively, these data demonstrate that the microbiota-derived metabolite IPA plays an important role in IAV infection.

## Discussion

The present study is the first to use shotgun metagenomics and targeted metabolomics to analyze the gut microbiota during experimental influenza. We believe that such an integrated multi-omics approach can provide a better understanding of the relationship between microbial changes and disease outcome. These analyses led us to identify IPA as an important metabolic modulator of the severity of influenza. In line with the results of previous studies of 16S rRNA amplicon sequencing in mouse,^9–14^ IAV infection induced alterations in the composition of the gut microbiota. Bacterial diversity diminished on D7, the peak of the acute phase. With some exceptions (see below), microbial variables returned to basal levels on D14. In IAV-infected mice (D7), the relative abundance of Bacillota and Actinomycetota was low (relative to controls), whereas that of the Verrucomicrobiota was considerably higher. It is noteworthy that although low abundances of Bacillota and Actinomycetota have been observed previously in patients with severe influenza,^18^ no such change has been reported for Verrucomicrobiota in humans. In the present study, more than 10 species were identified as being either significantly enriched or depleted during IAV infection. Strikingly (and in line with the 16S rRNA sequencing data^12,13^), *Akkermansia muciniphila* was the most enriched species on D7 and displayed normal levels on D14. *A. muciniphila* has recently emerged as a genus of major interest in health and disease.^36^ As *A. muciniphila* is a mucin degrader,^37^ one possible explanation for the observed enrichment is that influenza increased the levels of colonic mucin (as observed previously^38^), thus stimulating the growth of *A. muciniphila*. Notably, a reduction in oral feeding (a situation observed during experimental influenza^12^) is associated with an enhanced frequency of *A. muciniphila* in mice.^39^ The greater abundance of *A. muciniphila* during influenza infection is somewhat counterintuitive because this bacterium tends to be depleted in inflammatory settings and displays various beneficial anti-inflammatory and metabolic effects in both mice and humans.^24,40^ Although changes in *A. muciniphila* were not significantly correlated with the infectious and inflammatory markers examined in the present study, the transient bloom of this bacterium might have been a measure selected by the host to counter acute inflammation.^13^ Accordingly, supplementation with *A. muciniphila* during influenza (H7N9) reduced disease severity in mice.^13^ Given the known detrimental role of *A. muciniphila* in colitis and gut barrier functions,^39,41^ the bloom of this species might also contribute to some of the harmful effects associated with influenza, including intestinal inflammation and altered gut barrier properties^9,16^; this hypothesis requires further investigation (*e.g*. selective antibiotic treatment^39^). The most highly depleted species during the IAV infection belonged to the phylum Bacillota. We observed that the abundance of species, such as *L. taiwanensis*, *L. johnsonii* and *L. reuteri* (*Lactobacillaceae* and *Bifidobacteriaceae* families), was very low on D7. These bacterial species are considered beneficial to health and are currently used as probiotics.^42–44^ These species modulate local immune responses, enhance barrier function, and exert antibacterial activity against a broad spectrum of pathogenic bacteria. Notably, the relative abundance of these species was high on D14 (relative to D0), which suggests a long-term effect on disease outcomes. The levels of the acetate producer *B. animalis* spp. were low on D7 and D14. This probiotic species is positively associated with longevity in mice and negatively correlated with inflammation and obesity in humans.^45,46^ Our data are in line with Zhang et al.’s report of a positive association between *B. animalis* spp. levels and survival of IAV-infected mice.^14^ Of note, although the Bacteroidota species *Bacteroidales* bacterium M7 (a Gram-negative obligate anaerobe) was found to be enhanced on D7, shotgun sequencing did not reveal significant enrichments in the so-called “pathobionts” - including members of the *Alphaproteobacteria* and *Gammaproteobacteria* (*Escherichia* genus) classes. The latter result appears to contradict observations (including our previous work) of enrichment of these opportunistic bacteria during influenza.^9–12,18,20^ This disparity might be due to inter-study differences in experimental design, in the endogenous microbiota composition of the mice used, and the technique employed to study the composition of the gut microbiota:16S rRNA taxonomic profiling (with possible PCR-related bias and lower resolution) in previous studies and shotgun sequencing in the present study.

The metabolism of the gut microbiota plays a critical role in host physiology and immunity, including during infection.^12^ The effect of influenza on gut microbiota metabolism has not yet been fully characterized. To investigate this question, we combined a functional scale analysis of metagenomic data and targeted metabolomic analysis. Functional scale analysis predicted some interesting metabolic pathways affected by the virus, including those leading to the production of SCFAs. We have previously shown that IAV infection in mice is associated with a decrease in SCFAs production; however, the latter could not be attributed to a reduced number of reads for genes specifically involved in SCFA pathways.^12^ Indeed, our data suggest that changes in SCFA production were related to a lack of fiber intake by diseased animals (loss of appetite). Here, we observed changes in the pathways involved in SCFA metabolism, specifically those involved in butyrate production, an important metabolite in the context of influenza.^26,47,48^ Notably, these changes were associated with a decrease in the relative frequencies of the acetate producer *B. animalis* spp. (see the correlation analysis in Figure 5(d)) and the butyrate producer *Intestinimonas butyriciproducens* (with a decrease from 0.66% to 0.22% on D7), although the difference was not statistically significant (not shown). Notably, other typical SCFA producers, including *Lachnospiraceae* (*Lachnospiraceae* bacterium Spp and *Dorea* spp.) and *Ruminococcaceae* (*Pseudoflavonifractor capillosus*), had low abundance on D7, as previously observed in IAV-infected patients (not shown).^18,20^ “Lipopolysaccharide metabolism” was one of the most strongly enhanced metabolic pathway in infected animals. The apparent increase in LPS synthesis may be related to the greater abundance of gram-negative bacteria, such as members of the phylum Bacteroidetes. The intestinal barrier is disrupted during influenza,^16^ which suggests that LPS can diffuse into the blood and induce systemic inflammation and/or organ dysfunction. Other modules involved in lipid metabolism were altered during infection. We previously observed alterations in the metabolism of gut microbiota-derived glycosphingolipids, which are important in innate immunity during IAV infection.^49^ A change in the lipid metabolism of the gut microbiota during influenza was also observed in mice by Groves et al.^50^ who hypothesized that this was indirectly due to a loss of appetite. Other prominent metabolic pathway categories on D7 were “ATP synthesis” and “carbohydrate metabolism”. Bacterial genes involved in glycolysis and gluconeogenesis were also affected. Carbohydrate metabolism is known to provide energy (*e.g*. ATP) and precursors for many biosynthetic pathways, including SCFA synthesis. Hence, the low SCFA synthesis observed in IAV-infected mice is unlikely to be due to low levels of carbohydrate precursors. The enhancement of “carbohydrate metabolism” during infection might be due to greater mucus degradation by *A. muciniphila* (which uses mucus as an energy source) and might compensate for the lower food intake caused by loss of appetite. Furthermore, we observed influenza-related differences in metabolic pathways associated with amino acid synthesis. Although these changes did not translate into differences in blood amino acid concentrations (apart from non-significant trends for arginine and tryptophan), they might have consequences. In the gut, tryptophan is metabolized to several indole derivatives with important functions.^51,52^ Our shotgun metagenomic analysis also revealed a potential enhancement in the biosynthesis of polyamines during infection; the latter might be a reaction to the loss of appetite.^53^

Targeted metabolomic analysis confirmed changes in the predicted metabolic pathways. Major changes in blood metabolite concentrations were observed on D7, whereas most variables returned to basal levels on D14. We found that the metabolism of polyamine (on D7) and TMAO (on D14) was significantly increased during infection, whereas the synthesis of SCFAs and the tryptophan metabolite IPA was low. Low SCFA production was positively associated with a decrease in the abundance of Lactobacilli and *B. animalis* spp. and negatively associated with disease severity markers (e.g., pulmonary viral load). This finding is in line with those of other studies showing that SCFAs influence the host response to respiratory viruses.^26,47,48,54–56^ It is noteworthy that low SCFA production during influenza augments susceptibility to secondary bacterial infections in the lungs and gut.^12,16^ Given the pleiotropic functions of SCFAs, the decrease in SCFA levels during influenza may have other effects (e.g., systemic inflammation, metabolism, adipose tissue functions, and neuroprotection). Disruption of gut microbiota can alter the production of bacterial tryptophan metabolites. In particular, the level of the anti-inflammatory component IPA was very low on D7, which was concomitant with a reduction in the abundance of Lactobacillus species (including *L. reuteri*, a well-known commensal producer of indole derivatives).^57^ It is noteworthy that *Clostridium* and *Peptostreptococcu*s species also produce tryptophan metabolites, including IPA (for a review^58^). Importantly, the decrease in IPA levels correlated with the viral load and inflammation markers. The level of TMAO was low on D7, likely due to low food intake.^59^ In contrast, the TMAO levels were significantly elevated on D14. Given that the serum TMAO concentration is associated with many diseases ranging from inflammatory diseases (e.g., pneumonia) to metabolic and cardiovascular diseases,^28,60^ one can reasonably expect an elevated TMAO level can have negative consequences in IAV-infected hosts. Lastly, polyamines are anti-inflammatory and beneficial to human health. However, their role in lung diseases (including airway hyperresponsiveness) is less clear.^61,62^ The elevated concentration of polyamines (including spermidine) on D7 may be related to injury to the airway epithelium.^62^ Interestingly, the levels of putrescine, spermine, and spermidine were positively correlated with the viral load and inflammatory marker expression. Further functional analyses are required to establish a direct causal relationship between polyamine levels and disease severity in this influenza model. Taken together, the results of our integrated metagenomic and metabolomic analyses revealed major changes in the functional capacity of the gut microbiota during influenza and suggested that these changes have important consequences on disease outcomes.

Our metabolomics data showed that IPA was one of the most depleted microbiota-derived metabolites on D7 post-IAV infection. Therefore, we evaluated the functional consequences of IPA depression on influenza severity. Although the role of IPA during bacterial infections has been studied (mainly protection against sepsis),^63,64^ the putative effects of the compound during viral infections are more elusive. In a recent study, it was shown to abrogate the pathogenic effect of virus-specific cytotoxic T lymphocytes in certain strains of mice infected with lymphocytic choriomeningitis virus clone 13.^65^ The potential role of IPA in other viral infections, notably respiratory infections, remains unclear. This is a relevant question regarding the drop in IPA in HIV-infected people, a phenomenon usually associated with a change in the functional status of the gut microbiome.^66^ In our setting, IPA supplementation improved influenza outcomes (*e.g*., body weight, temperature) and translated into lower viral load and lower levels of local and systemic inflammation. As IPA supplementation had a low effect on the gut microbiota’s composition in our setting (at least at D7), it is unlikely that altered gut microbiota’s functionality due to IPA supplementation is causally linked with the beneficial effects described in this report.

In our settings, narrow-spectrum antibiotics and IPA supplementation suggest the involvement of IPA producers in influenza virus replication and inflammation. We are now intensively investigating IPA’s mode of action and strategies to optimize its beneficial effects on influenza outcomes. For instance, IPA mimetics^67^ may have enhanced beneficial effects during infection and decreased off-target effects relative to IPA. Modulation of gut microbiota and metabolites might be a potentially valuable strategy for the prevention and treatment of acute viral respiratory infections, including IAV and SARS-CoV-2 infections (for reviews^68,69^). Therefore, the use of prebiotics, probiotics, and postbiotics that can modulate the production of IPA and related metabolites is of potential interest. For example, supplementation with bacteria that produce IPA or with bacteria that modulate its production may limit the severity of influenza. Interestingly, *Clostridium sporogenes*, a bacterial species that produces IPA,^29^ has been used as a probiotic to reduce muscle cell inflammation.^70^ In conclusion, our results showed that IAV infection is associated with major changes in the functional status of the gut microbiota and highlighted IPA as an important contributor to influenza outcomes and potential biomarker of disease severity.

## Materials and methods

### Animals and ethics

Specific pathogen-free C57BL/6J mice (8-week-old, male) were purchased from Janvier (Le Genest-St-Isle, France). Mice were maintained in a biosafety level 2 facility at the Animal Resource Center at the Institut Pasteur de Lille for at least two weeks prior to use to allow appropriate acclimatization. Mice were fed standard rodent chow (SAFE A04) (SAFE, Augy, France) and water *ad libitium*. This diet contained 11.8% fiber, including 10% water-insoluble fiber (3.6% cellulose) and 1.8% water-soluble fiber. All experiments complied with current national and institutional regulations and ethical guidelines (Institut Pasteur de Lille/B59–350009). Protocols were approved by the regional Animal Experimentation Ethics Committee (Comité d’Ethique en Expérimentation Animale, Hauts de France, CEEA 75) and the French Ministry of Higher Education and Research (Ministère de l’Education Nationale, de l’Enseignement Supérieur et de la Recherche) (authorization numbers : 00357.03 and APAFIS 13,743–2018022211144403).

### Infection, supplementation with IPA and quantification of systemic cytokines

For infection, mice were anesthetized by intramuscular administration of 1.25 mg of ketamine and 0.25 mg of xylazine in 100 µl of phosphate buffered saline (PBS), and then intranasally (i.n.) infected with 50 μl of PBS containing 50 pfu of the H3N2 IAV strain A/Scotland/20/1974.^71,72^ Mice treated i. n. with PBS served as controls (non-infected mice). To study the impact of IPA treatment, mice were supplemented daily with IPA (Sigma-Aldrich, 40 mg/kg/day) or vehicle (0.5% methyl cellulose in sterile water) by oral gavage one day after infection with IAV and until D6 (sacrifice at D7 post-infection) or until D10 (sacrifice at D16 post-infection). The dose of IPA was chosen based on previous publications.^64,73^ Alternatively, mice were treated by oral gavage with IPA or vehicle one day before IAV infection until D3 (sacrifice at D4 post-infection). Each gavage dose volume was 200 μl. Body weight was monitored daily after IAV infection. To measure body temperature, mice were implanted subcutaneously with TAM-MT radio transmitters capable of monitoring body temperature through receivers (ReadStation, Intellebio innovation, Seichamps, France). Blood samples were collected at sacrifice, and cytokine levels were determined by enzyme-linked immunosorbent assay (ELISA) using kits provided by Invitrogen (Waltham, MA) and MyBioSource (San Diego, CA).

### Quantification of viral load by quantitative RT-PCR and immunofluorescence

Viral load in the lungs was determined by quantifying viral RNA encoding the M1 protein (segment 7).^67^ Briefly, after treatment with RNAse OUT (Invitrogen, Carlsbad, CA), RNA was reverse-transcribed with SuperScript® II Reverse Transcriptase (Invitrogen) using primers specific for M1 (5ʹ-TCT AAC CGA GGT CGA AAC GTA-3ʹ). qPCR was performed using TaqMan Universal PCR Master Mix (Applied Biosystems, Waltham, MA), using the following primers for M1 (forward:5ʹ-AAG ACC AAT CCT GTC ACC TCT GA-3ʹ; reverse:5ʹ-CAA AGC GTC TAC GCT GCA GTC C-3ʹ and M1 specific TaqMan probe (FAM) 5ʹ-TTT GTG TTC ACG CTC ACC GTG CC-3ʹ (TAMRA), and amplification was performed using the QuantStudio™ 5 K Flex Real-Time PCR System (Applied Biosystems). A synthetic gene containing the IAV M1 gene (segment 7) was used to construct a plasmid that was used to construct a standard curve. To analyze virus load by immunofluorescence, lung tissue sections (7 μm thick) were dried for 48 h at 42°C. The slides were rehydrated with toluene (AnalaR NORMAPUR ACS, VWR) at decreasing concentrations of ethanol in water. The rehydrated tissue sections were first treated with an antigen-unmasking solution (Tris EDTA buffer, pH 9). The sections were then rinsed and blocked for 1 h at room temperature in a blocking solution (PBS containing 5% BSA and 0.3% Triton X-100). Sections were incubated overnight at 4°C with a goat polyclonal anti-influenza A H3N2 (#PA1–7222, 1:1000, Invitrogen, Waltham, MA) antibody diluted in blocking solution. Sections were then washed and incubated at room temperature for 1 hour with Alexa Fluor-conjugated secondary antibodies (A-11037, 1:500) in the blocking solution. The coverslips were then mounted on slides using fluorescence mounting medium conjugated with DAPI (DAPI Fluoromount-G® #0100–20, SouthernBiotech, Birmingham, Al). Mounted slides were stored in the dark and at 4°C until image acquisition.

### Narrow spectrum antibiotic administration and supplementation of IPA

Depletion of IPA-producing bacteria was performed as described.^34,35^ Vancomycin (0.2 mg/ml) and ampicillin (0.5 mg/ml) were administered in drinking water five days prior to the onset of infection until D2. To study the impact of IPA treatment, mice were supplemented daily with IPA (40 mg/kg/day in 0.5% methyl cellulose) or vehicle by oral gavage one day before infection with IAV and until D3. To analyze the consequences of antibiotic treatment, with or without IPA supplementation, on viral load, mice were sacrificed at D4.

### Determination of gene expression using quantitative RT-PCR

Gene expression in the lungs was analyzed by quantitative RT-PCR, using standard procedures. RNA was reverse-transcribed using a High-Capacity cDNA Archive Kit (Life Technologies, Carlsbad, CA, USA). The resulting cDNA was amplified using SYBR Green-based real-time PCR and the QuantStudio™ 5 K Flex Real-Time PCR System (Applied Biosystems), according to the manufacturer’s protocol.^12^ The primers used are listed in Table 5. Relative mRNA levels were determined according to the 2^−ΔΔ^Ct (cycle thresholds) method by comparing (i) the PCR Ct for the gene of interest and the housekeeping gene (ΔCt) and (ii) the ΔCt values for the treated and control groups (ΔΔCt). Data were normalized against the expression of the *Gapdh g*ene and expressed as fold-change over the mean gene expression level in mock-treated mice.

**Table 5.** Sequences of the oligonucleotides usedin this study

*Gapdh*	Forward 5’-GCAAAGTGGAGATTGTTGCCA-3’	*Zo1*	Forward 5’-AGGTCTTCGCAGCTCCAAGAGAAA-3’
	Reverse 5’-GCCTTGACTGTGCCGTTGA-3’		Reverse 5’-ATCTGGCTCCTCTCTTGCCAACTT-3’
*Mx1*	Forward 5’-AGAAGGTGCGGCCCTGTATT-3’	*Foxj1*	Forward 5’-CCACCAAGATCACTCTGTCGG-3’
	Reverse 5’-TGAACTCTGGTCCCCAATGACA-3’		Reverse 5’-AGGACAGGTTGTGGCGGAT-3’
*Gbp2*	Forward 5’-AGGTTAACGGAAAACCCGTCA-3’	*Cc10*	Forward 5’-CATCTGCCCAGGATTTCTTCAA-3’
	Reverse 5’-CAGTCGCGGCTCATTAAAGCT-3’		Reverse 5’-CGCATTTTGCAGGTCTGAGC-3’
*Il1b*	Forward 5’-TCGTGCTGTCGGACCCATA-3’	*Ve-cadherin*	Forward 5’- −3’
	Reverse 5’-GTCGTTGCTTGGTTCTCCTTGT-3’		Reverse 5’- −3’
*Il6*	Forward 5’-CAACCACGGCCTTCCCTACT-3’	*Claudin-1*	Forward 5’-GGGGACAACATCGTGACCG-3’
	Reverse 5’-CCACGATTTCCCAGAGAACATG-3’		Reverse 5’-AGGAGTCGAAGACTTTGCACT-3’
*Cxcl2*	Forward 5’- GAAGTCATAGGCACTCTCA −3’	*Ifnb*	Forward 5’-TGGGTGGAATGAGACTATTGTTG-3’
	Reverse 5’- TTCCGTTGAGGGACAGCA −3’		Reverse 5’-CTCCCACGTCAATCTTTCCTC-3’
*Ifng*	Forward 5’-CAACAGCAAGGCGAAAAAG-3’	*Tnfa*	Forward 5’-CATCTTCTCAAAATTCGAGTGACA-3’
	Reverse 5’-GTGGACCACTCGGATGAGCT-3’		Reverse 5’-TGGGAGTAGACAAGGTACAACCC-3’
*Ccl2*	Forward 5’-GCAGCAGGTGTCCCAAAGAA-3’	*Ocln*	Forward 5’-AGCAGCCCTCAGGTGACTGTTATT-3’
	Reverse 5’-TCATTTGGTTCCGATCCAGGT-3’		Reverse 5’-ACGACGTTAACTCCTGAACAAGCA-3’

### Genomic DNA extraction and shotgun sequencing

To study the impact of IAV infection on the gut microbiota, cecal samples were collected from different sets of animals, including non-infected mice (sacrificed 7 days after PBS inoculation and termed D0) and IAV-infected mice (sacrificed 7 days and 14 days after virus inoculation, D7 and D14, respectively). Cecum homogenates were stored at −80°C until further analysis. Genomic DNA was extracted from cecal sample according to.^12^ Briefly, microbial DNA was extracted from approximately 150 mg of cecal samples (QIAamp Fast DNA Stool Mini Kit, QIAGEN, Stadt, Germany). Genomic DNA was purified using Agencourt AMPure XP magnetic beads (Beckman Coulter, Brea, CA, USA) and quantified according to GenoScreen protocol. The library preparation protocol was performed from 1ng of DNA (Nextera XT DNA Sample Prep Kit, Illumina, San Diego, CA, USA). The libraries were mixed with an equimolar amount of 2 nM. Shotgun sequencing was performed using a 250-bp paired-end sequencing protocol on an Illumina HiSeq 4000 platform (Illumina). The trimming and quality control of the data were performed using Fastp (version 0.20).^74^ The following default Fastp options were used: phred quality higher than or equal to Q15, percentage of bases allowed to be less than or equal to 40%, maximum number of N bases equal to 5. The argument “detect_adapter_for_pe” was used to allow adapter sequence trimming of paired-end reads, and the minimum read length (after adapter trimming) was 50 bp. Subsequent high-quality (HQ) reads were aligned to the mouse *Mus musculus* C57BL/6J genome assembly (NCBI assembly accession: GCF_000001635.26). The alignment was performed using Bowtie2 (version 2.3.5.1)^75^ with “–very-sensitive” and “–dovetail” parameters. Unaligned reads were retrieved using samtools (“-f 12 -F 256”). Unaligned HQ reads were used for downstream analyses. The total number of raw reads was between 119.846.552 and 50.895.518 (mean: 82.613.385; standard deviation (SD):18.227.130). The number of high-quality reads after decontamination ranged between 111.853.264 and 47.995.678 (mean:76.078.829, SD:16.762.266). The number of reads mapped on the iMGMC catalog ranged from 88.176.658 to 37.146.184 (mean:59.354.271; SD:13.281.256).

### Microbial community and taxonomic analyses

Alpha and beta diversity, multivariate analyses, and differential analysis were performed using the Vegan package (v2.5–7) in the R statistical environment (3.6.1). Alpha and beta diversities and multivariate analyses were performed up to the strain level. A Non-metric MultiDimensional Scaling plot (NMDS) using the Bray Curtis distance was constructed to assess beta diversity. A total of 10,000 permutations were performed for similarity and dissimilarity analysis^76^ and PERMANOVA. Differential analysis was performed separately from species to phylum taxonomic rank. Moreover, Kruskal-Wallis and Wilcoxon-Mann-Whitney tests were performed to highlight group effects. Statistical tests with p-values <0.05 were considered significant. The p-values were corrected using the Benjamini-Hochberg procedure to control for the false discovery rate. For differential abundance analysis, the R package Deseq2 v1.34.0. Taxonomic abundance was estimated using mOTUs v2.6.0 with default parameters.^77^ In short, mOTUs2 is based on universal, reference-independent, phylogenetic marker gene (MG)-based operational taxonomic units (*i.e*., called mOTUs), enabling profiling of microbial species in shotgun sequencing data. LEfSe was used to identify the organisms most likely to explain differences between days of infection.^78^ LEfSe was executed in its Galaxy module, with an alpha value for the factorial Kruskal-Wallis test among classes of 0.05, and a threshold on the logarithmic LDA score of 2.0 * *. The results are visualized in a bar plot of the descending LDA score per class.

### Functional analysis and estimation of KOs counts and abundances

Functional analysis was performed using the murine gut microbiome catalog, iMGMC.^79^ Reads were mapped against the 4.9 M genes of the iMGMC catalog. Alignments were considered valid when the forward and reverse reads were mapped to the same gene catalog with the correct orientation. The resulting raw gene counts were normalized and translated into relative abundance in a sample-wise manner. Normalization factors were computed using the function calcNormFactors, with the trimmed mean of M-values (TMM) method from edgeR. TMM normalization has been shown to perform well on shotgun metagenomic data with a low false positive rate.^80^ Raw counts were divided by the product of the library size and the normalization factor and then by gene length (RPKM). Relative abundances were determined by dividing by the sample-wise sum of all normalized counts. Relative abundances of iMGMC genes were translated into KOs abundances-like using the KofamScan results available from the iMGMC GitHub repository. Genes lacking KO correspondence were summed into a single non-annotated feature. KOs counts were computed similarly, but from raw gene counts.

### Computation of KEGG modules abundances

KEGG module definitions were used to estimate their abundance in each sample. Briefly, KEGG modules are subdivided into steps, each defined as a sequence of AND (*e.g*., protein complexes) and OR (*e.g*. several KOs can perform the same reaction) operations. To estimate the abundance of modules from the rules, the AND and OR operations were replaced by MIN and MAX, respectively, resulting in a single abundance-like metric for each step. The pseudo-abundances of the modules were then computed as the mean of the abundances across all module steps. If more than half of a module’s steps harbored a null abundance, the abundance of the module was set to 0. To test for differential abundance of modules between groups, Wilcoxon and Kruskal-Wallis tests (R, package rstatix) were performed. The resulting p-values were adjusted using the Benjamini-Hochberg procedure.

### Computation of GOmixer modules abundances for short-chain fatty acids

To compute the module abundances of metabolic pathways involved in short-chain fatty acid synthesis and degradation, GOmixer v1.7.5.0 (Raes Lab, Ghent, Belgium) was used. GOmixer is a human gut-specific metabolic pathway analysis tool (available as an online tool and downloadable software package at http://www.raeslab.org/gomixer/). It quantifies human gut metabolic pathway modules by mapping KOs abundance on a database of predefined gut-specific modules. A module is a set of highly related enzymatic functions that represents a cellular process with defined input and output metabolites. The modules used in the GOmixer database were manually compiled based on literature searches.

### Detection of inter-group varying KOs and aggregation at the pathway level

The KOs varying between the groups were estimated using the Songbird multinomial algorithm.^81^ Raw KO counts (including non-annotated features) were used as the inputs. A different model was computed for each combination of groups (namely D0-D7, D0-D14 and D7-D14 albeit we only present the results for the D0-D7 and D7-D14 models). KOs with fewer than 10 counts for any combination were excluded from the input table for songbirds. The parameters’ epochs“and differential-prior” were set to 5000 and 0.5, respectively. In each model, a coefficient (also called the differential in GitHub) was computed for each KO. To present the songbird results at the pathway level (Figure 3(c)), 1000 KOs (25% of the roughly 4000 KOs) with the most extreme coefficients were selected in each model independently. Then, for each module, the coefficients of the KOs taking part were summed to obtain an overview of the global trend in the module. The modules were then grouped into categories according to their biological function. Thus, each point in the plot represents the sum of the coefficients in the module. The lines are the medians and boxes Q1 and Q3, respectively.

### Metabolomic analysis

A targeted quantitative approach was implemented to analyze the mouse serum samples. This method was based on the MxP^Ⓡ^ Quant 500 kit (Biocrates, Innsbruck, Austria) using Flow Injection Analysis (FIA) and LC-MS/MS. The assay kit enabled quantification of 630 metabolites. FIA was used for the semi-quantitative measurement of 523 hydrophobic molecules such as acylcarnitines. A total of 107 metabolites (amino acids, indoles, etc.) were measured by high-performance liquid chromatography. This technique uses isotope-labeled internal standards and provides quantitative results based on calibration curves and rigorous quality control analysis (QCs). Briefly, 10 μL of serum samples were loaded onto a filter paper and dried in a stream of nitrogen for derivatization with a solution of 5% phenyl isothiocyanate. Subsequently, the dried residues were extracted with methanol containing 5 mM ammonium acetate. The analysis was performed on a QTRAP 5500 System (Sciex, Framingham, MA, USA) with an FIA method or coupled to a UFLC-20XR (Shimadzu, Kyoto, Japan) using a column provided in the kit. Multiple reaction monitoring was used for the quantification. MetIDQ software (Biocrates) was used to calculate the concentrations of individual metabolites. The experiments were validated using calibration curves and quality-control protocols. For each metabolite, the peaks were quantified using the area under the curve. Metabolites containing more than 10% of missing values per group (< LLOQ, < LOD) were discarded. Missing values (one value maximum out of eight) were replaced with the median of the respective groups.

### Quantification of tryptophan metabolites by LC-MS/MS

An analytical protocol based on targeted LC-MS/MS was developed to measure tryptophan metabolites. The latter (with tryptophan) includes 5-hydroxytryptophan, serotonin, kynurenin, 3-hydroxy kynurenin, 3-hydroxy anthranilic acid, xantherunic acid, kynurenic acid, quinaldic acid, 8-hydroxy quinaldic acid, anthranilic acid, indol-3-acetic acid, tryptamine, indole-3-lactic acid, 5-hydroxy indole-3-acetic acid, quinolinic acid, picolinic acid, indole-3-carboxaldehyde, and indol-3-propionic acid. Serum or cecal content (50 µL) was mixed with 50 µL pure acetonitrile (for protein precipitation) containing deuterated compounds at 50 μ µM as an internal standard (CDN isotopes, Pointe-Claire, QC, Canada). The supernatant (50 µL) was then added to deionized water (600 µL). Ten microliters of this mixture were injected onto an UPLC-MS/MS system (Acquity TQ-XS Detector, Waters, Milford, MA) equipped with a C18-XB column (1.7 µm-100Å-150 × 2.1 mm from Phenomenex®, Torrance, USA). The ions of each analyzed compound were detected in positive ion mode using multiple reaction monitoring. The Masslinks software (Waters) was used for data acquisition and processing.

### Correlation analysis

For metabolites, missing values were replaced by the median of the respective groups (one value maximum on 8). Table preparation and filtering of the results were conducted using Python’s library Pandas.^82,83^ The tables were then passed in a pairwise manner as input for Hierarchical All-against-All association (HAllA),^84^ a framework for the calculation of pairwise correlation, hierarchical clustering, and the identification of densely associated blocks. For HAllA execution, the parameters were specified as follows: Spearman similarity metric, false discovery rate (FDR) control using the Benjamini-Hochberg method with alpha 0.05, and the default expected false negative rate (FNR) for detection of densely associated blocks of 0.2. The heatmap-based representation hallagram provided by HAllA was used for the visualization. The associations calculated by HallA were integrated into a single table, filtered for an adjusted p-value ≤0.05, and correlation coefficient ρ ≥ |0.15| and visualized.

### 16S rRNA sequencing and gut microbiota analysis

To determine the impact of IPA and vancomycin and ampicillin supplementation during influenza, the gut microbiota’s composition was analyzed by 16S rRNA sequencing. Briefly, a set of 12 primers (forward) and 3 primers (reverse) were used to amplify the V3 and V4 hypervariable region of the 16S rRNA gene fragment using an optimized 16S-amplicon-library preparation protocol (Biomnigene, Besançon France). 16S rRNA gene PCR was performed using 5 µl of 1/40 diluted genomic DNA using GoTaq® Rapid PCR Master Mix (Promega, Madison, WI) using 15 barcoded primers at final concentrations of 0.2 μM and an annealing temperature of 55°C for 38 cycles. The PCR products were multiplexed at equal concentrations and purified using a PippinHT system (Sage Science, Beverly, MA). Sequencing was performed using a 250-bp paired-end sequencing protocol on an Illumina MiSeq platform (Illumina, San Diego, CA). A step of removal of low-quality reads from the raw paired-end reads were performed using Fastp. Remaining sequences were assigned to samples based on barcode matches using cutadapt (version 4.4). Data were then imported in Qiime2 (2023.7) and forward and reverse primer sequences were removed using the cutadapt plugin. The sequences were denoized using the DADA2 method, and reads were classified using the GTDB database (release 214). For the determination of the impact of IPA supplementation during influenza, a total of 449,106 paired-end reads were analyzed, with an average of 17,964 per sample (range: 6,162 to 27,728). For the determination of the impact of ampicillin and vancomycin supplementation during influenza, a total of 225,000 paired-end reads were analyzed, with an average of 7,300 per sample (range: 1625 to 18,181). Beta diversity was computed using Qiime2. Bray Curtis-based PCA was performed to assess beta diversity. Differences between groups were tested using PERMANOVA analysis. Raw sequence data (effect of IPA) are accessible in the National Center for Biotechnology Information (project number PRJNA1054515), biosample accession numbers SAMN38923322 to SAMN38923345 (https://dataview.ncbi.nlm.nih.gov/object/PRJNA1054515?reviewer=s9vludrl2tgbq9smpruea58tu2). Differential analysis was performed using the LEfSe pipeline.

### Statistical analyses

For infectious markers, results are expressed as mean ± SD unless otherwise stated. Statistical analyses were performed using GraphPad Prism v8.0.2 and R v4.0.2 softwares. The Mann-Whitney *U* test was used to compare the two groups, unless otherwise stated. Comparisons of more than two groups were analyzed using the one-way ANOVA Kruskal-Wallis test (nonparametric), followed by Dunn’s posttest. *p < 0.05; ** *p* < 0.01, *** *p* < 0.001.

## Supplementary Material

Supplemental Material

## Data Availability

The sequence datasets generated in this study are publicly available at https://www.ncbi.nlm.nih.gov/bioproject/PRJNA908759 and https://www.ncbi.nlm.nih.gov/bioproject/PRJNA105451. iMGMC (https://zenodo.org/record/3631711 and https://github.com/tillrobin/iMGMC). The file used was called “GeneID,” and the KOs annotations are available from the github (KEGG KofamScan 03/20). Reference genome (for decontamination): Assembly GCF_000001635.26, available at https://www.ncbi.nlm.nih.gov/assembly/GCF_000001635.26/.

## Abbreviations

IAV, influenza A virus; LBP, lipopolysaccharide-binding protein; SCFAs, short-chain fatty acids; RT-PCR, reverse transcription polymerase chain reaction; IL, interleukin; TMAO, trimethylamine N-oxide; IPA, indole-3-propionic acid; IAA, indole-3-acetic acid.
