# Supplementary figures and images for: Shotgun metagenomics and systemic targeted metabolomics highlight indole-3-propionic acid as a protective gut microbial metabolite against influenza infection
Source: Gut Microbes. 2024 Mar 6;16(1):2325067. doi: 10.1080/19490976.2024.2325067 (PMC10936607; doi:10.1080/19490976.2024.2325067)

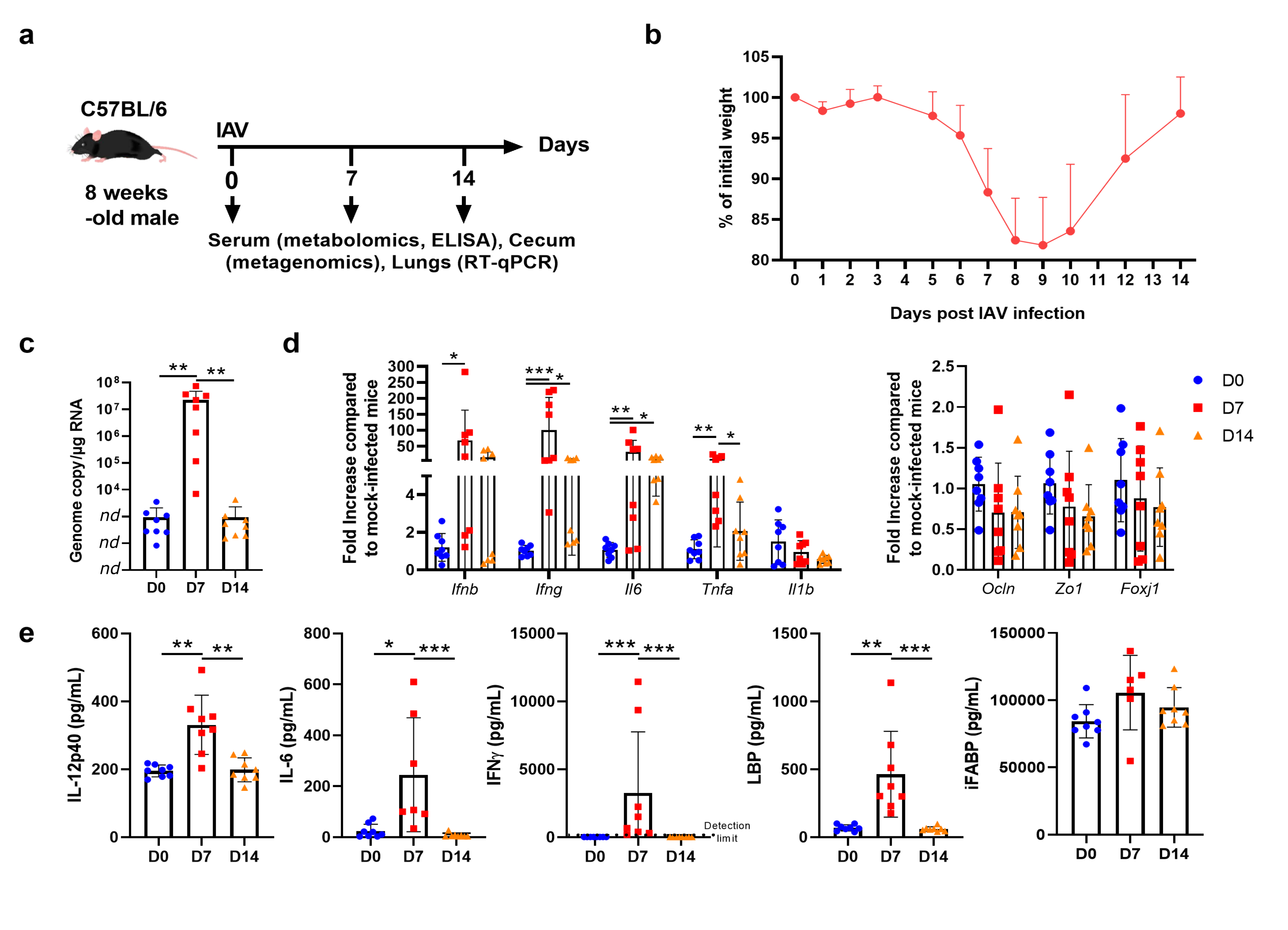

Supplement: Supplemental Material [file KGMI_A_2325067_SM4607.zip › Sup Figure 1 revised.tiff]

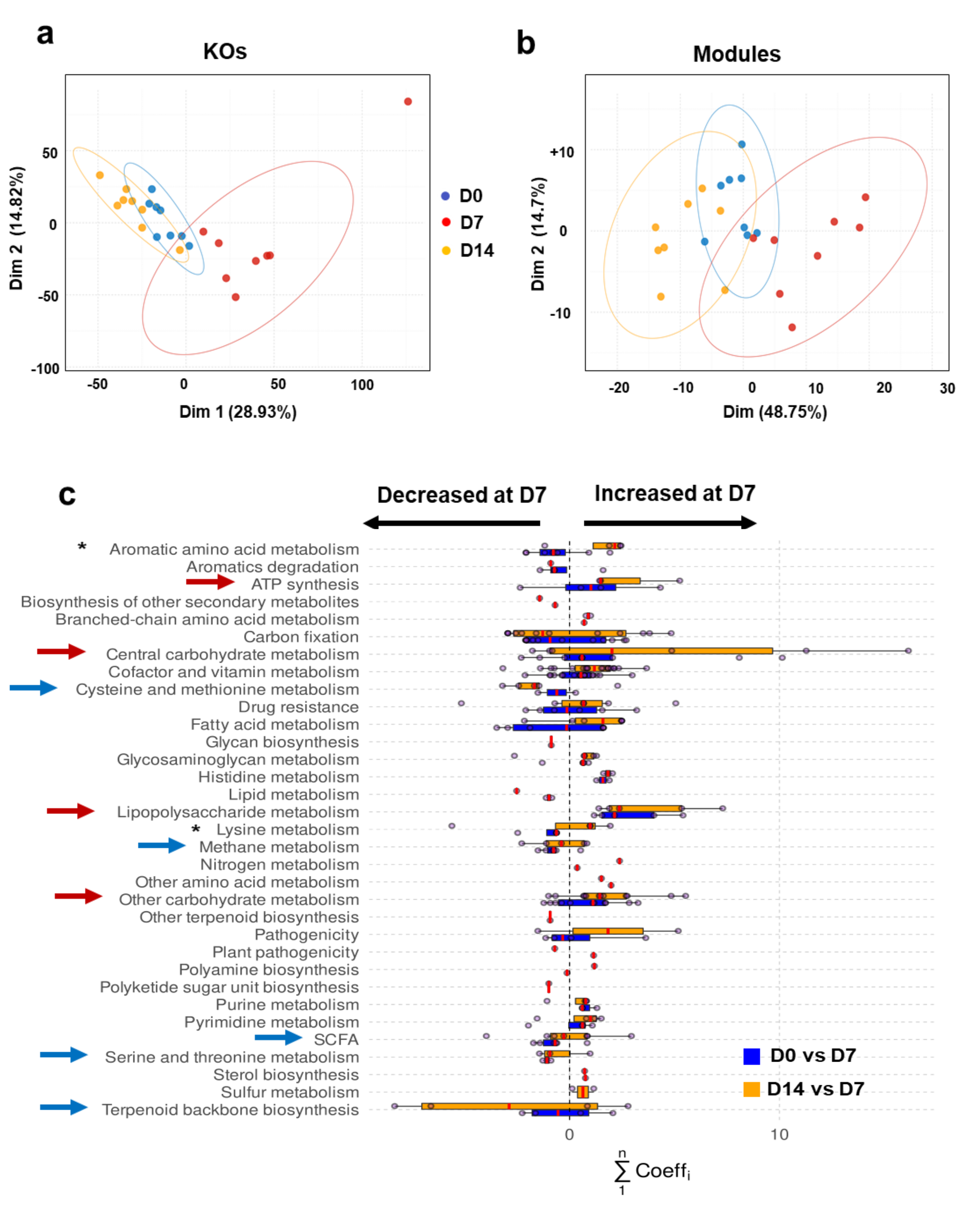

Supplement: Supplemental Material [file KGMI_A_2325067_SM4607.zip › Supplemental Figure 2.tiff]

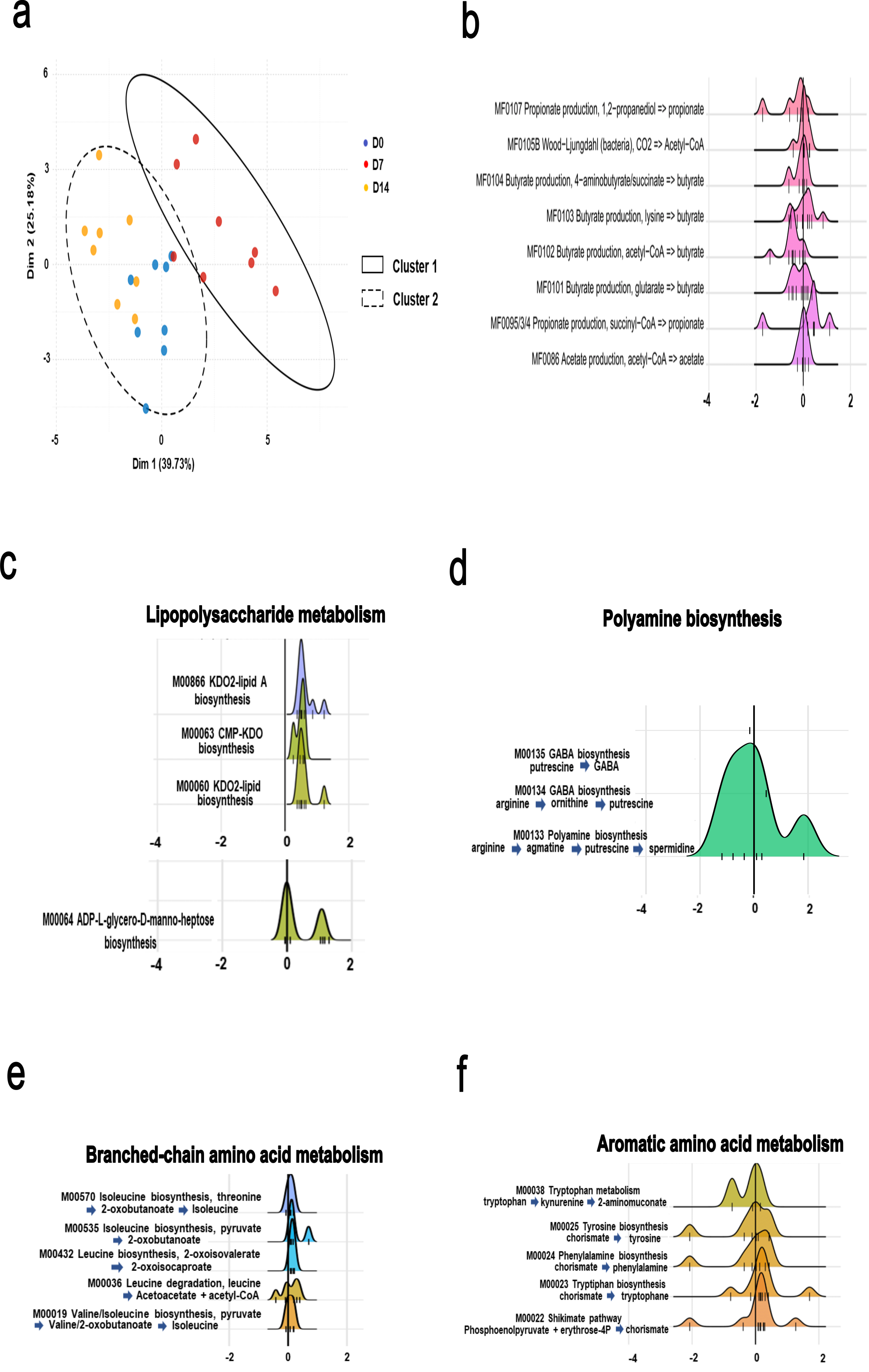

Supplement: Supplemental Material [file KGMI_A_2325067_SM4607.zip › Supplemental Figure 3.tiff]

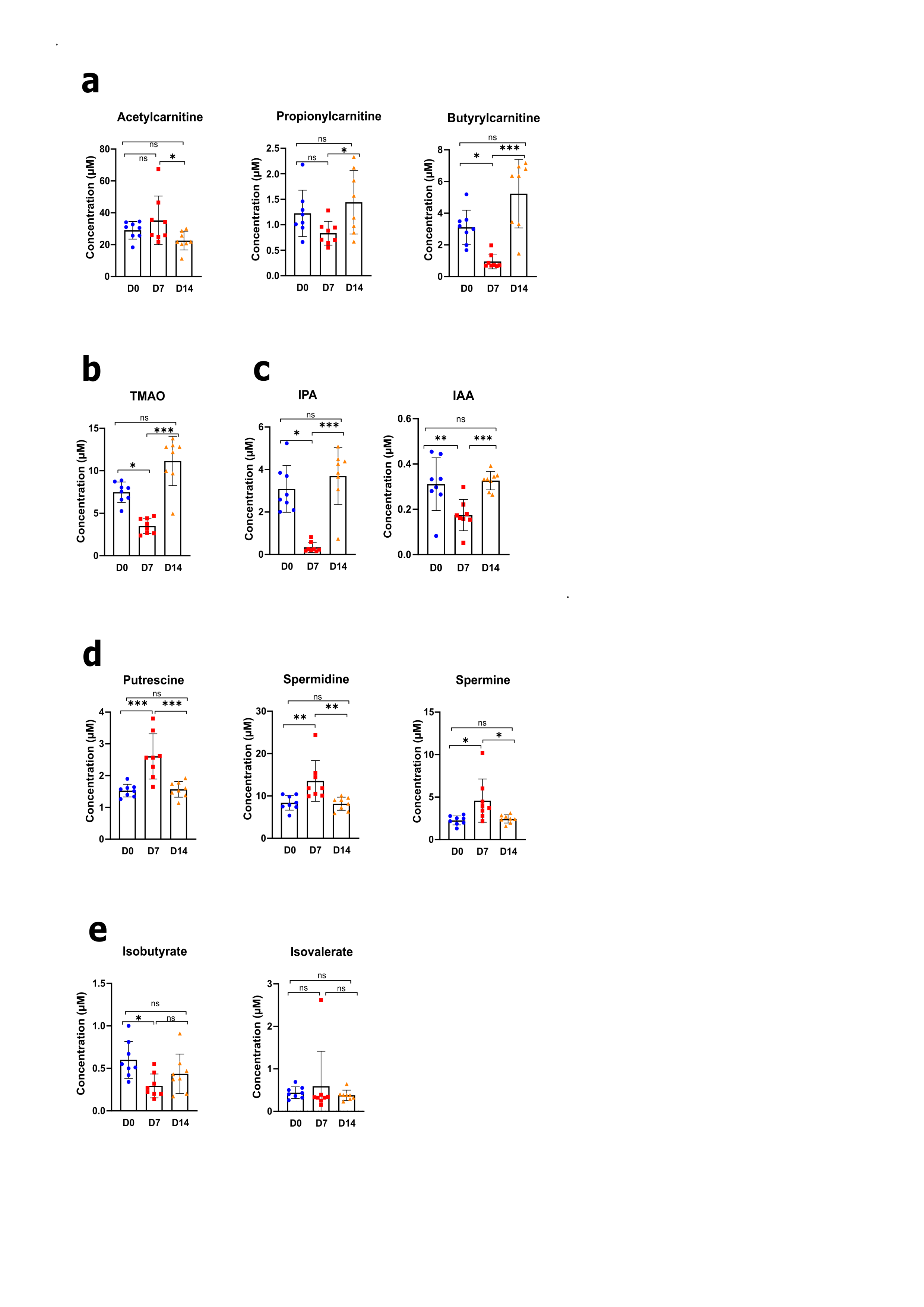

Supplement: Supplemental Material [file KGMI_A_2325067_SM4607.zip › Supplemental Figure 4.tiff]

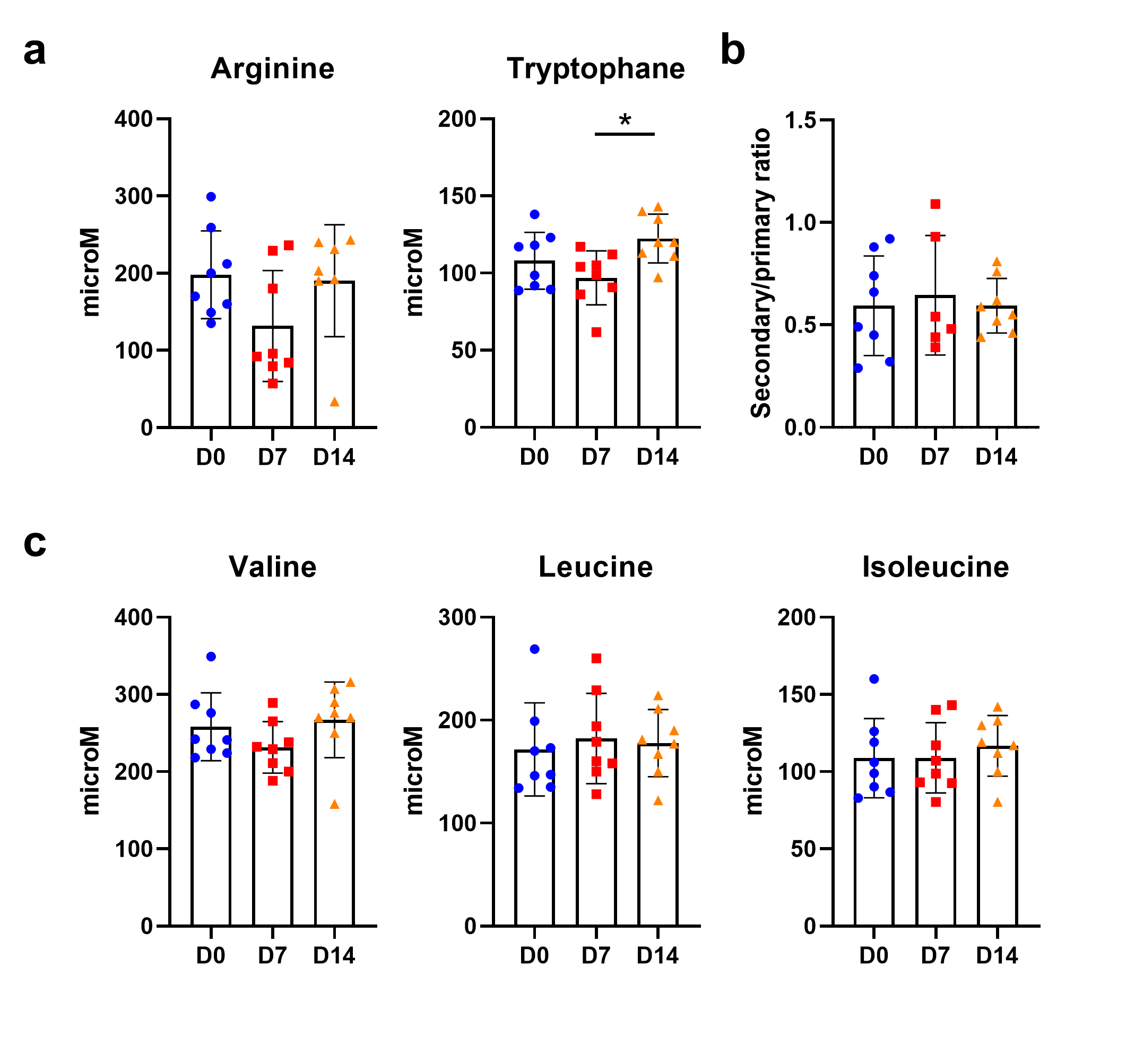

Supplement: Supplemental Material [file KGMI_A_2325067_SM4607.zip › Supplemental Figure 5.tif]

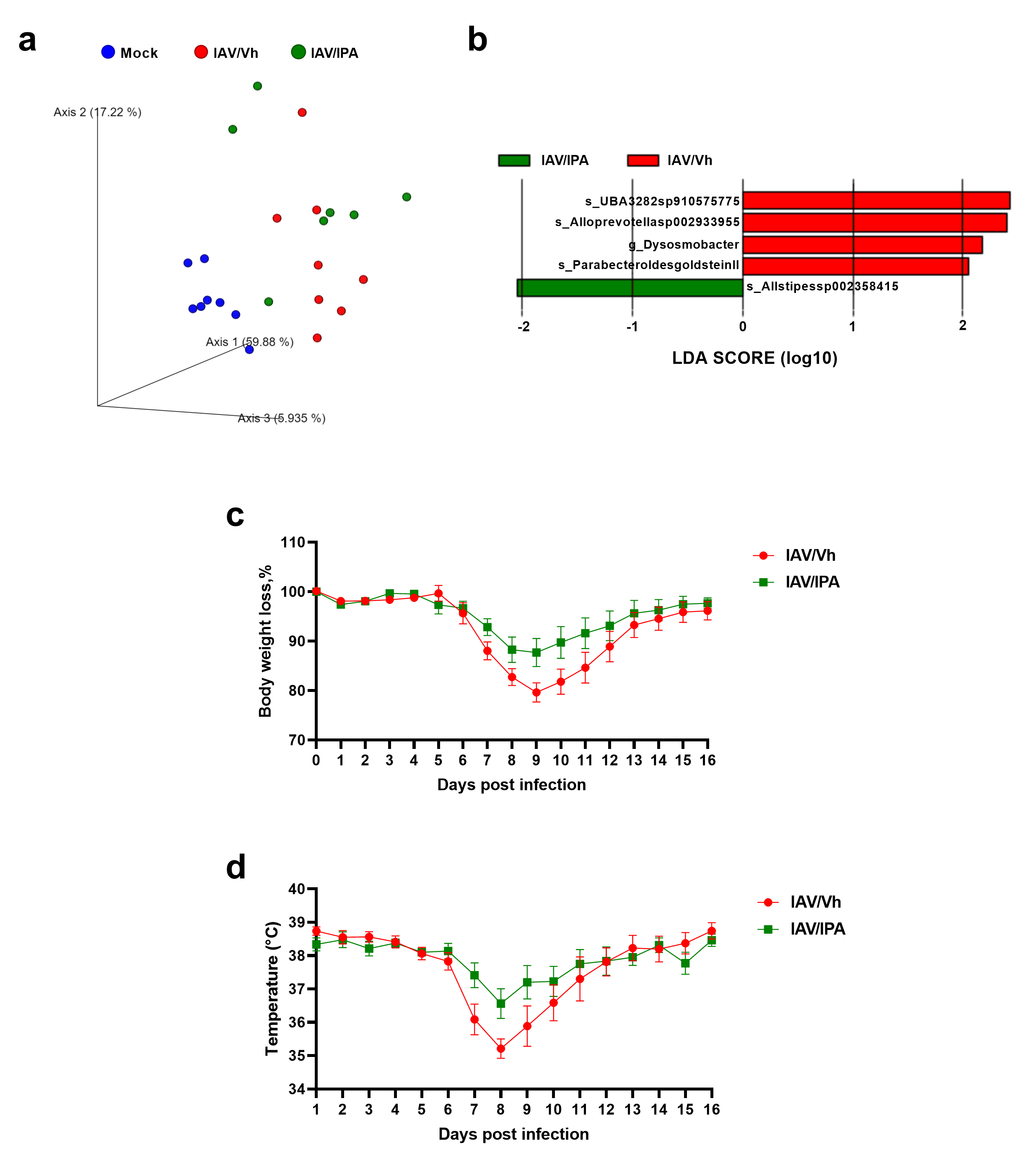

Supplement: Supplemental Material [file KGMI_A_2325067_SM4607.zip › Supplementary Figure 6.tif]

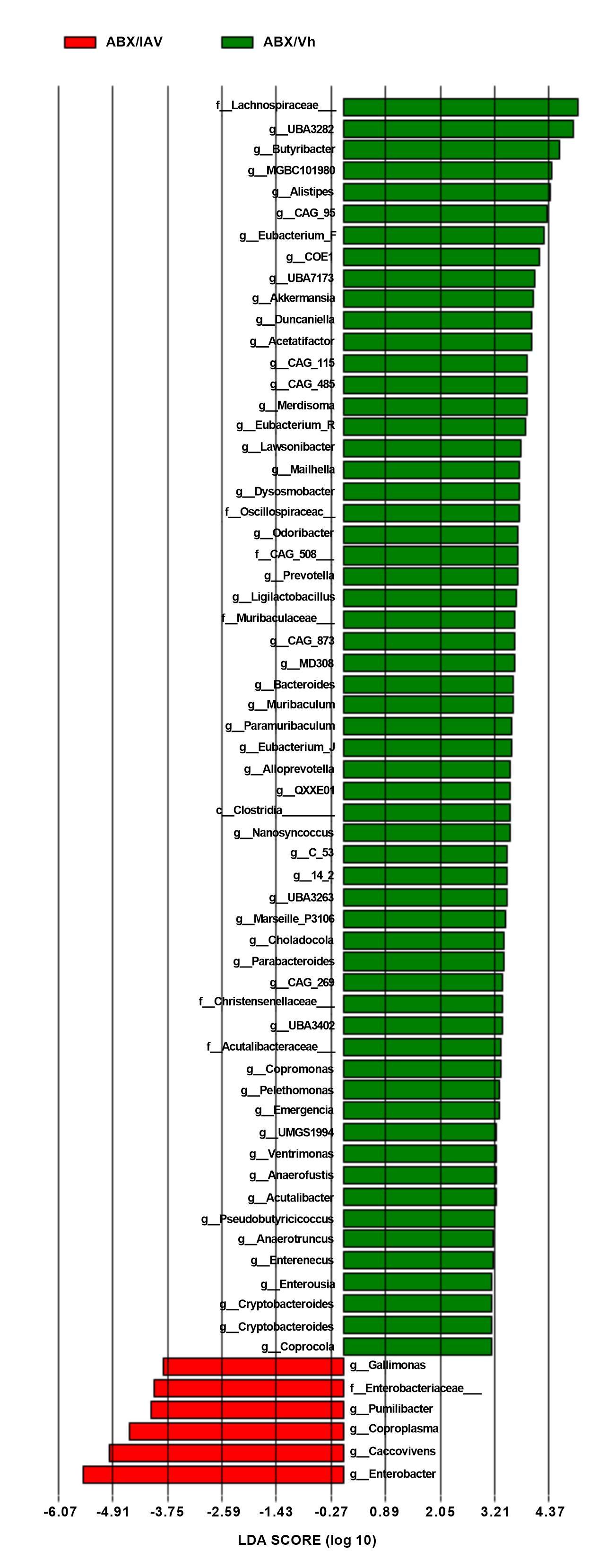

Supplement: Supplemental Material [file KGMI_A_2325067_SM4607.zip › Supplementary Figure 7.tif]
